# An Analytical Framework for Phenotypic Selection of Fitness-Conferring Genes

**DOI:** 10.1007/s11538-026-01670-y

**Published:** 2026-05-29

**Authors:** Marc Sturrock, Anna Sturrock

**Affiliations:** Department of Physiology and Medical Physics, Royal College of Surgeons in Ireland, Dublin, Ireland

**Keywords:** Phenotypic selection, Stochastic gene expression, Selective enrichment, Translational feedback, Fluctuation–response relation, Drug resistance

## Abstract

Phenotypic selection can cause the transient, selective upregulation of fitness-conferring genes in isogenic cell populations under stress, producing selective enrichment of the fitness gene relative to a neutral reference gene. While computational models have shown that such enrichment requires noisy gene expression and a cellular memory linking growth rate to gene expression (Ciechonska et al. 2022), the precise mechanistic requirements and the analytical principles governing enrichment have remained unclear. Here, we present an exact analytical framework that unifies enrichment mechanisms across both growth-driven and death-driven selection regimes. By analysing a stochastic model of explicit mRNA and protein dynamics, we prove that when selection acts via cell division, the fitness advantage of faster growth is exactly cancelled by the penalty of faster protein dilution. We show that this cancellation is bypassed by translational feedback but not by transcriptional feedback alone; for genes with regulated (switching) promoters, the promoter-state memory provides an independent route to enrichment without translational feedback. Conversely, when selection acts via cell death, this exact cancellation is bypassed, allowing selective enrichment to emerge from baseline gene expression noise without any assumptions about growth-related feedback loops or regulated vs constitutive expression. We derive an exact fluctuation–response relation demonstrating that, in all cases, enrichment scales with the super-Poissonian component of unperturbed protein noise times the relevant memory timescale. All analytical predictions are corroborated by stochastic simulations of a finite-population Moran model. These results have implications for the emergence of drug resistance: by transiently enriching survival-conferring phenotypes, phenotypic selection can extend the window during which cell division occurs under stress, increasing the opportunity for permanent genetic mutations to arise.

## Introduction

Genetically identical cell populations can exhibit phenotypic heterogeneity due to stochastic fluctuations in gene expression (Elowitz et al. 2002; Swain et al. 2002). Under environmental stress, this heterogeneity provides raw material for phenotypic selection: cells that happen to express higher levels of a fitness-conferring gene divide more frequently or survive longer, and thereby increase in relative abundance within the population. This leads to an apparent upregulation of the fitness gene at the population level, a phenomenon we term “selective enrichment” in this paper (Tsuru et al. 2011; Ciechonska et al. 2022).

Selective enrichment is distinct from genetic mutation, signal transduction, and bistable switching. It can operate even on a monostable, constitutively expressed gene, relying solely on the interplay between stochastic gene expression and fitness-dependent selection (via differential growth or survival). A natural way to quantify selective enrichment is to compare the fitness gene against a reference gene expressed with identical parameters in the same cell but whose protein does not affect the cell’s fitness. Since both genes have identical expression parameters and share the same intracellular environment, the ratio $\langle n \rangle /\langle r \rangle$ of their mean protein levels isolates the effect of selection: any deviation from unity is a direct signature of selective enrichment. Understanding the mechanisms of selective enrichment is important because, although phenotypic selection is transient and non-genetic, it can extend the survival of subpopulations under stress, providing additional rounds of cell division during which permanent genetic resistance mutations may arise. This positions phenotypic selection as a potential stepping stone toward heritable drug resistance, both in bacterial antibiotic treatment and in chemotherapy applied to cancer cells (Ciechonska et al. 2022).

The analytical foundations for understanding phenotypic selection on gene expression were laid by Mora and Walczak (2013), who studied a birth–death model for protein copy number *n* with an *n*-dependent growth rate (denoted *s*(*n*) in their notation, not to be confused with the selection strength parameter *s*). For linear selection $s(n) = sn$, they showed that the steady-state distribution remains Poisson but with a shifted mean $\langle n \rangle = b/(d-s)$, where *b* is the protein production rate and *d* is the degradation rate. Compared to a reference gene unaffected by selection ($\langle r \rangle = b/d$), this gives a ratio $\langle n \rangle /\langle r \rangle = d/(d-s) > 1$ for positive selection, demonstrating selective enrichment.

However, the Mora–Walczak model treats protein dilution via a constant effective degradation rate *d*, independent of the cell’s growth rate. In reality, protein dilution occurs at cell division, when molecules are partitioned between daughter cells: a cell that grows faster divides sooner and dilutes its proteins more rapidly. This coupling between growth rate and dilution has important consequences for selective enrichment, as we demonstrate below (Huh and Paulsson 2011; Thomas and Shahrezaei 2021).

Computational agent-based models incorporating explicit cell division, stochastic gene expression, and various biological feedbacks were used to systematically investigate selective enrichment (Ciechonska et al. 2022). That study identified two key ingredients: variability in gene expression within an isogenic population, and a cellular “memory” from positive feedbacks between growth and expression. More specifically, their systematic comparison of ten candidate models revealed that, for growth-driven selection, enrichment required: (i) explicit modelling of both mRNA and protein (a two-stage model), (ii) growth-rate-dependent dilution, and (iii) a global positive feedback coupling the translation rate to the cell growth rate. Notably, a model with a growth-rate-dependent transcription rate but a growth-rate-independent per-mRNA translation rate (Model 6 of Ciechonska et al. 2022) did not produce selective enrichment, and Bayesian model comparison assigned the model with growth-dependent translation alone (Model 7) as most likely to produce enrichment, as opposed, perhaps counter-intuitively, to the model with both transcriptional and translational feedbacks (Model 8).

In this paper, we provide an analytical theory that explains these computational findings. We begin by proving two no-enrichment theorems for growth-coupled dilution (Theorems 1 and 2), demonstrating that when protein dilution is coupled to the growth rate but there is no translational feedback, the ratio $\langle n \rangle /\langle r \rangle = 1$ exactly, for any selection strength $s \ge 0$. This holds regardless of whether transcriptional feedback is present, and requires no moment closure approximation. We then extend the model to include explicit mRNA dynamics and a global translational feedback (translation rate scales with growth rate), showing that selective enrichment emerges. For a general model with transcriptional feedback $q_1$ and translational feedback $q_2$, the enrichment ratio at weak selection is

1 $$\begin{aligned} \frac{\langle n \rangle }{\langle r \rangle } \approx 1 + \frac{s\,q_2}{d_m + 2\mu _0}, \end{aligned}$$

which is independent of $q_1$ at leading order. To generalise this result, we derive an exact (all-orders) relation showing that selective enrichment is proportional to $q_2$ times the covariance asymmetry $\textrm{Cov}(n, m_n) - \textrm{Cov}(n, m_r)$. The underlying mechanism can be understood in terms of timescales: translational feedback exploits the mRNA autocorrelation time $\tau _m = 1/(d_m + \mu _0)$ to amplify gene-specific stochastic memory. The intrinsic covariance $C_{nm} = \textrm{Cov}(n, m_n)$ between a protein and its own mRNA (Thattai and van Oudenaarden 2001; Paulsson 2005) provides the memory window; translational feedback converts this memory into a selective advantage because it multiplies the gene’s own mRNA ($m_n$ or $m_r$). Transcriptional feedback, by contrast, acts upstream of the gene-specific mRNA and creates no gene-specific memory that persists across cell division events. We further show that when selection acts via differential cell death rather than differential growth, this exact cancellation is bypassed entirely, and selective enrichment emerges from baseline gene expression noise without any translational feedback. All analytical predictions are corroborated by stochastic simulations of a finite-population Moran model.

## Model Framework

We work within the population-level framework of Mora and Walczak (2013). A population of cells is described by the density $\rho _{\textbf{x}}(t)$, where $\textbf{x}$ denotes the intracellular state of a cell. Within each cell, biochemical reactions govern the stochastic dynamics of gene expression.

To keep the population size constant (as in a chemostat or turbidostat; Gresham and Dunham 2014), each cell division displaces a randomly chosen cell, creating an effective death rate equal to the population-mean growth rate $\langle \mu \rangle$. The normalised probability $P_{\textbf{x}}(t) = \rho _{\textbf{x}}(t)/\sum _{\textbf{x}'} \rho _{\textbf{x}'}(t)$ then satisfies

2 $$\begin{aligned} \frac{\partial P_{\textbf{x}}}{\partial t} = \mathscr {L}[P]_{\textbf{x}} + \big (\mu (\textbf{x}) - \langle \mu \rangle \big ) P_{\textbf{x}}, \end{aligned}$$

where $\mathscr {L}$ is the operator describing intracellular birth–death dynamics.

Each cell contains two genes: a fitness gene producing protein *n* and a reference gene producing protein *r*. Both genes are constitutively expressed with identical parameters. The cell’s growth rate depends only on the fitness protein: $\mu (n) = \mu _0 + sn$, where $\mu _0$ is the basal growth rate and $s > 0$ quantifies the selection strength. In Section 3.6, we generalise this framework to include death-driven selection, where the fitness protein reduces a cell’s death rate rather than increasing its growth rate.

In Models A–E below, both genes are constitutively expressed (no promoter regulation); we relax this assumption in Section 3.5. We consider five models of increasing biological realism.

### Model A: Mora–Walczak Baseline (Protein only, Constant Dilution)

Protein dynamics follow a simple birth–death process with constant rates. There is no explicit mRNA. Proteins are produced at a constant rate *b* and degraded at rate *d* per molecule, giving a total degradation rate $d \cdot n$ per cell. The growth rate is $\mu (n) = sn$. This is the model studied by Mora and Walczak (2013). Dilution is absorbed into *d*, independent of growth rate.

### Model B: Growth-Coupled Dilution (Protein only, No Feedback)

The per-molecule dilution rate equals the cell’s growth rate. There is no explicit mRNA. Proteins are produced at a constant rate *b* and diluted at rate $\mu (n)$ per molecule, giving a total dilution rate $\mu (n) \cdot n$ per cell. The growth rate is $\mu (n) = \mu _0 + sn$.

### Models C–E: Growth-Coupled Dilution with Explicit mRNA and Feedback

We now include explicit mRNA dynamics and allow for two types of global positive feedback: growth-dependent transcription (strength $q_1$) and growth-dependent translation (strength $q_2$). These are motivated by evidence that both RNA polymerase abundance and ribosome abundance scale with growth rate in bacteria (Klumpp and Hwa 2008; Klumpp et al. 2009; Dai et al. 2016; Shahrezaei and Marguerat 2015).

For each gene (*n* or *r*), the intracellular reactions with growth rate $\mu (n) = \mu _0 + sn$ are:

$$\begin{aligned}&{\text {Transcription:}}&\emptyset&\rightarrow m &  {\text {at rate }} k_m + q_1 \mu (n), \\&{\text {mRNA degradation/dilution:}}&m&\rightarrow m-1 &  {\text {at rate }} (d_m + \mu (n)) \cdot m, \\&{\text {Translation:}}&m&\rightarrow m + {\text {protein}} &  {\text {at rate }} (k_p + q_2 \mu (n)) \cdot m, \\&{\text {Protein dilution:}}&{\text {protein}}&\rightarrow {\text {protein}} - 1 &  {\text {at rate }} \mu (n) \cdot {\text {protein}}. \end{aligned}$$

Three special cases are of interest, corresponding directly to Models 6–8 of Ciechonska et al. (2022): Model C (transcriptional feedback only, $q_1 > 0$, $q_2 = 0$), Model D (translational feedback only, $q_1 = 0$, $q_2 > 0$), and Model E (both feedbacks, $q_1 > 0$, $q_2 > 0$).

### General Moment Equation

For any intracellular quantity $\phi (\textbf{x})$, the evolution of its population mean under Eq. (2) is

3 $$\begin{aligned} \frac{d\langle \phi \rangle }{dt} = \sum _{{\text {reactions}}} \langle {\text {rate}} \times \Delta \phi \rangle + s \cdot \textrm{Cov}(n, \phi ), \end{aligned}$$

where $\Delta \phi$ is the change in $\phi$ due to each reaction. With linear selection $\mu (n) = \mu _0 + sn$, the selection contribution takes the form $s\,\textrm{Cov}(n, \phi ) = s(\langle n\phi \rangle - \langle n \rangle \langle \phi \rangle )$, a manifestation of Fisher’s fundamental theorem (Fisher 1930). A detailed derivation, starting from Eq. (2), is provided in Appendix A.

Eq. (2) describes a population in which the only intracellular state variable that enters selection is the protein copy number *n*, and in which cell age and cell size do not appear explicitly. There is a more detailed master equation framework for agent-based models of cell populations with explicit age (or cell size), developed by Thomas (2017) and Thomas and Shahrezaei (2021). In that framework, cells are tracked as individuals with an age *a* (or size *V*) as well as an intracellular state, and the population density satisfies a master equation that couples the intracellular reaction dynamics to deterministic ageing or growth and to stochastic division. For traits that do not themselves affect the division rate, Thomas (2017) established an ergodic principle stating that the statistics of cell histories, obtained by tracing a cell in the population back to the ancestor from which it originated, coincide with those of an age-sorted population snapshot; Thomas and Shahrezaei (2021) analysed the related coupling of gene-expression noise with cell size in this agent-based setting. Rather than tracking age or size, Eq. (2) follows the population-averaged intracellular dynamics directly, with an effective dilution rate equal to the population mean growth rate. The no-enrichment theorems below are accordingly statements about population-level means, which is the level of description relevant to selective enrichment; the age- or size-resolved frameworks of the cited works address the complementary question of how individual cell histories progress through the cell cycle. We return to the connection with single-cell lineage data, and the fitness-landscape inference of Nozoe et al. (2017), in Section 3.3 and the Discussion.

### Model A: The Mora–Walczak Result

Applying Eq. (3) to $\phi = n$:

4 $$\begin{aligned} \frac{d\langle n \rangle }{dt} = b - d\langle n \rangle + s\,\textrm{Var}(n). \end{aligned}$$

Since the steady-state distribution is Poisson ($\textrm{Var}(n) = \langle n \rangle$) (Mora and Walczak 2013), the steady state gives

5 $$\begin{aligned} \langle n \rangle = \frac{b}{d - s}, \qquad \langle r \rangle = \frac{b}{d}, \qquad \frac{\langle n \rangle }{\langle r \rangle } = \frac{d}{d-s} > 1. \end{aligned}$$

This is the main result of Mora and Walczak (2013): selection effectively reduces the degradation rate from *d* to $d-s$, producing selective enrichment.

## Results

### Growth-Coupled Dilution Exactly Cancels Selection

#### Theorem 1

(No enrichment under growth-coupled dilution) Consider a protein produced at constant rate *b* and diluted at the growth rate $\mu (n)$, where $\mu$ is any function of the fitness protein copy number *n*. Let the reference protein *r* be produced at the same rate *b* and diluted at the same growth rate $\mu (n)$. Then $\langle n \rangle /\langle r \rangle = 1$ at steady state, for any fitness landscape $\mu (n)$.

#### Proof

We first prove the result for linear selection $\mu (n) = \mu _0 + sn$, then generalise. Applying Eq. (3):

6 $$\begin{aligned} \frac{d\langle n \rangle }{dt}&= \underbrace{b}_{{\text {production}}} - \underbrace{\langle (\mu _0 + sn) \cdot n \rangle }_{{\text {dilution}}} + \underbrace{s\,\textrm{Var}(n)}_{{\text {selection}}} \nonumber \\&= b - \mu _0\langle n \rangle - s\langle n^2 \rangle + s(\langle n^2 \rangle - \langle n \rangle ^2) = b - \mu _0\langle n \rangle - s\langle n \rangle ^2. \end{aligned}$$

The $\langle n^2 \rangle$ terms from dilution and selection cancel exactly, leaving a closed equation for $\langle n \rangle$ that requires no moment closure. The steady state satisfies

7 $$\begin{aligned} s\langle n \rangle ^2 + \mu _0\langle n \rangle - b = 0, \qquad \langle n \rangle = \frac{-\mu _0 + \sqrt{\mu _0^2 + 4sb}}{2s}. \end{aligned}$$

For the reference protein, the same cancellation yields

8 $$\begin{aligned} \frac{d\langle r \rangle }{dt} = b - (\mu _0 + s\langle n \rangle )\langle r \rangle , \qquad \langle r \rangle = \frac{b}{\mu _0 + s\langle n \rangle }. \end{aligned}$$

Using $b = \langle n \rangle (\mu _0 + s\langle n \rangle )$ from Eq. (7):

9 $$\begin{aligned} {\frac{\langle n \rangle }{\langle r \rangle } = 1 \quad {\text {(exactly, for all }} s \ge 0 \text {).}} \end{aligned}$$

The cancellation does not rely on the linear form $\mu (n) = \mu _0 + sn$. For a general fitness landscape $\mu (n)$, the general moment equation (Appendix A) gives

10 $$\begin{aligned} \frac{d\langle n \rangle }{dt} = b - \langle \mu (n)\cdot n \rangle + \big (\langle \mu (n)\cdot n \rangle - \langle \mu \rangle \langle n \rangle \big ) = b - \langle \mu \rangle \langle n \rangle , \end{aligned}$$

where the dilution term $-\langle \mu (n)\cdot n \rangle$ and the selection term $+\langle \mu (n)\cdot n \rangle - \langle \mu \rangle \langle n \rangle$ annihilate the $\langle \mu (n)\cdot n \rangle$ dependence exactly for any $\mu (n)$. An identical argument gives $d\langle r \rangle /dt = b - \langle \mu \rangle \langle r \rangle$. Therefore $\langle n \rangle /\langle r \rangle = 1$ for any nonlinear fitness landscape. $\square$

**Fig. 1:**
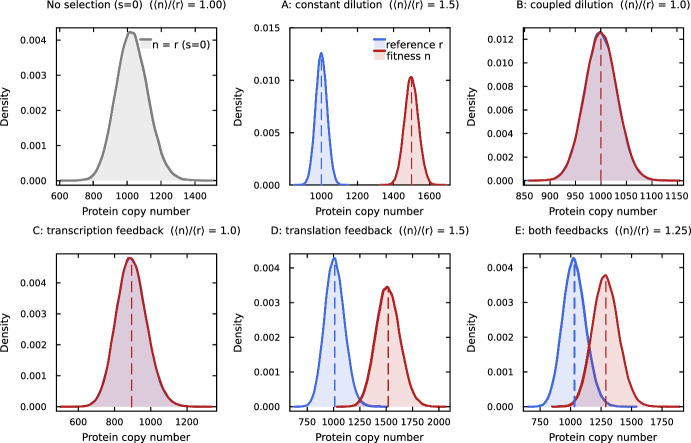
Distributional view of selective enrichment across the five base models (Models A–E, see Table 1) at fixed selection strength $s = 0.5$. Each panel shows kernel density estimates of the protein copy number distribution for the fitness gene *n* (red) and reference gene *r* (blue), with dashed vertical lines at the population means $\langle n \rangle , \langle r \rangle$ and the enrichment ratio $\langle n \rangle /\langle r \rangle$ annotated in each panel title. Curves are analytical distributions matched to the first two moments of each model: negative binomials for the two-stage models C–E (with shape parameters set from the Gaussian moment closure of Appendix D), and Poissons for the protein-only models A and B. Model A (constant dilution) produces a clear rightward shift of the fitness distribution relative to the reference; Models B and C (growth-coupled dilution, with or without transcriptional feedback) show perfect overlap ($\langle n \rangle /\langle r \rangle = 1$, in agreement with Theorems 1 and 2); Models D and E (translational feedback) restore the shift, with Model E slightly suppressed relative to Model D (Eq. (30)). The top-left panel shows the baseline distribution at $s=0$, identical for the fitness and reference genes in all models. Parameters for the two-stage models C–E: $k_m = 110$, $k_p = 40$, $d_m = 4$, $\mu _0 = 1$, with $q_1 = 2$ and $q_2 = 6$ where the respective feedback is present. Models A and B are protein-only; Model A uses a constant degradation rate $d_A = 1.5$ in place of growth-coupled dilution

Growth-coupled dilution completely eliminates selective enrichment: the selective advantage of faster-growing cells is exactly offset by their faster protein dilution. Figure 1 provides a distributional view of these results, showing how the protein copy number distributions for the fitness and reference genes differ across models; the top-right panel (Model B) shows the perfect overlap predicted by Theorem 1.

### Translational Feedback is Necessary and Sufficient for Selective Enrichment

We now analyse the general model with mRNA and both feedbacks ($q_1$, $q_2$).

#### mRNA Means

Applying Eq. (3) to $\phi = m_n$:

11 $$\begin{aligned} \frac{d\langle m_n \rangle }{dt}&= \langle k_m + q_1(\mu _0 + sn) \rangle - \langle (d_m + \mu _0 + sn)m_n \rangle + s\,\textrm{Cov}(n, m_n) \nonumber \\&= (k_m + q_1\mu _0) + q_1 s\langle n \rangle - (d_m + \mu _0)\langle m_n \rangle - s\langle nm_n \rangle \nonumber \\ &\quad + s(\langle nm_n \rangle - \langle n \rangle \langle m_n \rangle ) \nonumber \\&= (k_m + q_1\mu _0 + q_1 s\langle n \rangle ) - (d_m + \mu _0 + s\langle n \rangle )\langle m_n \rangle . \end{aligned}$$

The raw cross-moment $\langle nm_n \rangle$ cancels exactly. Note that the transcription feedback appears only through $\langle n \rangle$, which is the same for both genes. Therefore an identical equation holds for $\langle m_r \rangle$, and at steady state:

12 $$\begin{aligned} \langle m_n \rangle = \langle m_r \rangle = \frac{k_m + q_1\mu _0 + q_1 s\langle n \rangle }{d_m + \mu _0 + s\langle n \rangle } \equiv m^*. \end{aligned}$$

The mRNA means are always equal for the two genes, regardless of the transcriptional feedback strength $q_1$.

#### Protein Means

For the fitness protein *n*, with translation rate $(k_p + q_2(\mu _0 + sn)) \cdot m_n$:

13 $$\begin{aligned} \frac{d\langle n \rangle }{dt}&= \langle (k_p + q_2\mu _0 + q_2 sn)m_n \rangle - \langle (\mu _0 + sn)n \rangle + s\,\textrm{Var}(n) \nonumber \\&= (k_p + q_2\mu _0)\langle m_n \rangle + q_2 s\langle nm_n \rangle - \mu _0\langle n \rangle - s\langle n \rangle ^2. \end{aligned}$$

For the reference protein *r*:

14 $$\begin{aligned} \frac{d\langle r \rangle }{dt}&= (k_p + q_2\mu _0)\langle m_r \rangle + q_2 s\langle nm_r \rangle - (\mu _0 + s\langle n \rangle )\langle r \rangle . \end{aligned}$$

The key structural difference lies in the cross-moment terms. For the fitness protein, the term $q_2 s\langle nm_n \rangle$ involves *n* and its own mRNA $m_n$, whereas for the reference protein, the term $q_2 s\langle nm_r \rangle$ involves *n* and the reference mRNA $m_r$. These terms are present if and only if $q_2 \ne 0$. When $q_2 = 0$ (no translational feedback), both protein equations become closed (requiring no higher-order moments) and reduce to the form of Model B.

#### Model C: Transcriptional Feedback only ($q_1 > 0$, $q_2 = 0$)

##### Theorem 2

(No enrichment under transcriptional feedback) Consider the two-stage model with growth-coupled dilution and an arbitrary transcription function $h(\mu , V)$ of the growth rate and the cell volume, but with a growth-rate-independent translation rate ($q_2 = 0$). Then $\langle n \rangle /\langle r \rangle = 1$ at steady state, for any fitness landscape $\mu (n)$ and any transcription function *h*.

##### Proof

Setting $q_2 = 0$, the protein equations become:

15 $$\begin{aligned} \frac{d\langle n \rangle }{dt}&= k_p\langle m_n \rangle - \mu _0\langle n \rangle - s\langle n \rangle ^2, \end{aligned}$$

16 $$\begin{aligned} \frac{d\langle r \rangle }{dt}&= k_p\langle m_r \rangle - (\mu _0 + s\langle n \rangle )\langle r \rangle . \end{aligned}$$

Since $\langle m_n \rangle = \langle m_r \rangle$, these have identical source terms. By the same algebraic argument as Theorem 1:

17 $$\begin{aligned} {\frac{\langle n \rangle }{\langle r \rangle } = 1 \quad {\text {(exactly, for all }} s \ge 0 {\text {, any }} q_1 \ge 0 \text {).}} \end{aligned}$$

The transcription rate $(k_m + q_1\mu (n))$ depends on *n* through the growth rate, but it does not multiply any gene-specific stochastic variable. The production of mRNA for gene *n* and gene *r* is affected identically by the growth-rate-dependent transcription enhancement, so the mRNA means remain equal and the downstream protein dynamics inherit this symmetry. For a general transcription function $h(\mu )$, the mRNA source $\langle h(\mu (n)) \rangle$ is always gene-independent, ensuring $\langle m_n \rangle = \langle m_r \rangle$ and hence $\langle n \rangle /\langle r \rangle = 1$ regardless of the form of $\mu$ or *h*. The same conclusion holds when the transcription rate also depends on the cell volume, $h(\mu , V)$. The growth rate and the cell volume are both cell-global quantities, shared by the fitness and reference genes within a cell, so a volume-dependent transcription rate produces mRNA for the two genes at identical rates and the source $\langle h(\mu (n), V) \rangle$ remains gene-independent. In particular, the size-scaling of transcription observed for many eukaryotic genes (Padovan-Merhar et al. 2015; Sun et al. 2020), in which the bulk transcription rate increases with cell volume, cannot by itself generate selective enrichment; as in the growth-rate-dependent case, enrichment requires a feedback that multiplies a gene-specific stochastic variable, which is the role played by the translation rate in Models D and E. $\square$

This result explains why Model 6 of Ciechonska et al. (2022), which had growth-dependent transcription but no translational feedback, produced a max ratio of exactly 1.0 in computational simulations. The bottom-left panel of Figure 1 (Model C) illustrates this: the fitness and reference distributions overlap perfectly despite the presence of transcriptional feedback.

#### Models D and E: Translational Feedback ($q_2 > 0$)

When $q_2 > 0$, the protein equations contain the unclosed cross-moments $\langle nm_n \rangle$ and $\langle nm_r \rangle$. We proceed by perturbative expansion in *s* (weak selection).

#### Zeroth Order ($s = 0$)

At zero selection, the growth rate is constant ($\mu _0$) and both genes are independent and identically distributed. The effective rates are:

18 $$\begin{aligned} k_m^{{\text {eff}}} = k_m + q_1\mu _0, \qquad k_p^{{\text {eff}}} = k_p + q_2\mu _0, \end{aligned}$$

and the steady-state values are:

19 $$\begin{aligned} m_0 = \frac{k_m^{{\text {eff}}}}{d_m + \mu _0}, \qquad n_0 = \frac{k_p^{{\text {eff}}} m_0}{\mu _0}. \end{aligned}$$

#### Intrinsic Protein–mRNA Covariance

The covariance between a protein and its own mRNA at $s = 0$, computed from the Lyapunov equation (Appendix B), is

20 $$\begin{aligned} C_{nm} \equiv \textrm{Cov}(n, m_n)\big |_{s=0} = \frac{k_p^{{\text {eff}}} m_0}{d_m + 2\mu _0}. \end{aligned}$$

This is always positive: cells with more mRNA of a gene tend to have more of that gene’s protein. Importantly, the fitness protein *n* and the reference mRNA $m_r$ are independent at $s=0$:

21 $$\begin{aligned} \textrm{Cov}(n, m_r)\big |_{s=0} = 0. \end{aligned}$$

#### First-Order Corrections

Expanding $\langle n \rangle = n_0 + sn_1$, $\langle r \rangle = n_0 + sr_1$, and similarly for mRNA means, the first-order steady-state conditions give (see Appendix C for details). For the fitness protein, the *O*(*s*) balance of Eq. (13) is

22 $$\begin{aligned} \mu _0\,n_1 = k_p^{{\text {eff}}} m_1 + q_2\,(C_{nm} + n_0 m_0) - n_0^2. \end{aligned}$$

For the reference protein, using $\textrm{Cov}(n, m_r)|_{s=0} = 0$:

23 $$\begin{aligned} \mu _0\,r_1 = k_p^{{\text {eff}}} m_1 + q_2\,n_0 m_0 - n_0^2. \end{aligned}$$

The terms $q_2 n_0 m_0 - n_0^2$ are common to both equations and cancel in the difference. The transcriptional feedback $q_1$ enters only through $m_0$ and $m_1$, which are identical for both genes.

#### The Enrichment Ratio

Subtracting Eqs. (22) and (23):

24 $$\begin{aligned} \mu _0(n_1 - r_1) = q_2\,C_{nm}, \qquad n_1 - r_1 = \frac{q_2\,C_{nm}}{\mu _0} = \frac{q_2\,k_p^{{\text {eff}}}\,m_0}{\mu _0(d_m + 2\mu _0)} > 0. \end{aligned}$$

Dividing by $n_0 = k_p^{{\text {eff}}} m_0/\mu _0$, the ratio of means is

25 $$\begin{aligned} {\frac{\langle n \rangle }{\langle r \rangle } \approx 1 + \frac{s\,q_2}{d_m + 2\mu _0}.} \end{aligned}$$

This is our main finding for the growth-dependent enrichment analysis. At leading order in *s*, the enrichment ratio is completely independent of the transcriptional feedback strength $q_1$; only $q_2$ (translational feedback) appears. The ratio reduces to unity when $q_2 = 0$, consistent with the exact results for Models B and C. The Fano factor of the two-stage model at $s = 0$ is $F = 1 + k_p^{{\text {eff}}}/(d_m + 2\mu _0) = 1 + \beta$, where $\beta = k_p^{{\text {eff}}}/(d_m + 2\mu _0)$ is the effective translational burst contribution (the ratio of the effective translation rate to the total mRNA turnover rate, incorporating growth-rate corrections to both). Writing $\alpha = q_2/k_p^{{\text {eff}}}$, we have $q_2/(d_m + 2\mu _0) = \alpha \beta$, and the enrichment ratio equals $1 + s\alpha (F-1)$: selective enrichment scales directly with the super-Poissonian component of protein noise.

#### Exact Relation at Finite Selection

Although Models D and E give identical enrichment ratios at first order in *s*, an exact (all-orders) relation reveals their structural difference. Subtracting the steady-state equations (13) and (14), and using $\langle m_n \rangle = \langle m_r \rangle = m^*$, yields

26 $$\begin{aligned} {(\mu _0 + s\langle n \rangle )(\langle n \rangle - \langle r \rangle ) = q_2\,s\,\big (\textrm{Cov}(n, m_n) - \textrm{Cov}(n, m_r)\big ).} \end{aligned}$$

This relation holds exactly, without perturbative expansion or moment closure. It shows that the enrichment difference $\langle n \rangle - \langle r \rangle$ is proportional to $q_2$ at all orders in *s*, so that if $q_2 = 0$ the right-hand side vanishes and Theorems 1 and 2 are recovered immediately. The driving force is the covariance asymmetry $\Delta C \equiv \textrm{Cov}(n, m_n) - \textrm{Cov}(n, m_r)$, which measures how much more strongly the fitness protein correlates with its own mRNA than with the reference mRNA. At $s = 0$, $\textrm{Cov}(n, m_n) = C_{nm}$ and $\textrm{Cov}(n, m_r) = 0$, so $\Delta C = C_{nm}$ and the first-order result (25) is recovered. At finite *s*, both covariances shift; the effect of $q_1$ on these covariance shifts is discussed below in the context of the signal-to-penalty decomposition.

#### Second-Order Correction: Transcriptional Feedback Suppresses Selective Enrichment

Although $q_1$ is absent at first order, the exact relation (26) reveals how it enters at the next order. Rewriting Eq. (26) as

27 $$\begin{aligned} \frac{\langle n \rangle }{\langle r \rangle } - 1 = \frac{q_2\,s\,\Delta C}{(\mu _0 + s\langle n \rangle )\,\langle r \rangle }, \end{aligned}$$

and approximating $\Delta C \approx C_{nm}$ (its value at $s = 0$, valid at leading order), $\langle n \rangle \approx \langle r \rangle \approx n_0$, gives

28 $$\begin{aligned} \frac{\langle n \rangle }{\langle r \rangle } \approx 1 + \frac{s\,q_2}{d_m + 2\mu _0} \cdot \frac{1}{1 + s\,n_0/\mu _0}, \end{aligned}$$

where the zeroth-order protein level

29 $$\begin{aligned} n_0 = \frac{k_p^{{\text {eff}}}(k_m + q_1\mu _0)}{\mu _0(d_m + \mu _0)} \end{aligned}$$

grows linearly with the transcriptional feedback strength $q_1$. The factor $1/(1 + s\,n_0/\mu _0)$ represents a nonlinear dilution penalty: at finite selection, the growth rate $\mu _0 + s\langle n \rangle$ exceeds the basal rate $\mu _0$, accelerating dilution. Transcriptional feedback inflates $n_0$ (by boosting mRNA production via $k_m + q_1\mu _0$), which amplifies this penalty without proportionally strengthening the gene-specific signal $\Delta C$ in the numerator. The cancellation of $q_1$ at first order occurs because $C_{nm} \propto n_0$ (both scale with $k_m + q_1\mu _0$), so the ratio $C_{nm}/n_0$ is $q_1$-independent. At second order, however, the denominator grows as $n_0^2$ while the numerator grows only as $n_0$, and the suppression becomes visible.

Expanding Eq. (28) to second order in *s*:

30 $$\begin{aligned} \frac{\langle n \rangle }{\langle r \rangle } \approx 1 + \frac{s\,q_2}{d_m + 2\mu _0} - \frac{s^2\,q_2\,n_0}{\mu _0(d_m + 2\mu _0)} + O(s^3). \end{aligned}$$

**Fig. 2:**
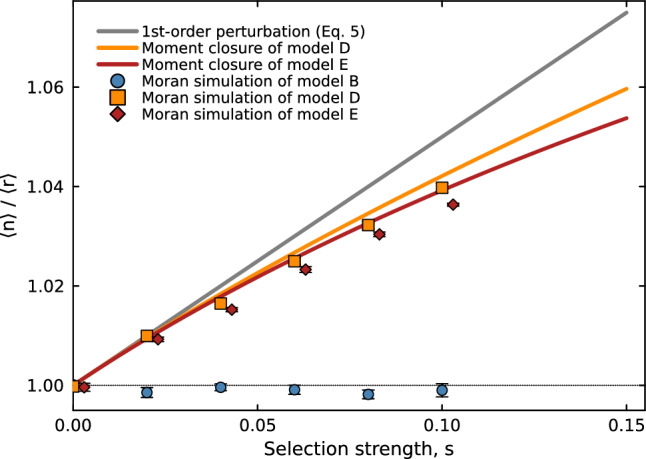
Enrichment ratio $\langle n \rangle /\langle r \rangle$ as a function of selection strength *s*: validation of the analytical predictions against finite-population Moran simulations. The first-order perturbation result (grey solid line, Eq. (25), valid for $s n_0 / \mu _0 \ll 1$) and the non-perturbative Gaussian moment closure (solid coloured lines, one per model; Appendix D) are compared with Moran-model simulation means ($N = 3000$ cells, $T = 300$ hr per replicate, 10 independent replicates, error bars are $\pm 1$ SE). Model B (circles, growth-coupled dilution, no feedback) remains at $\langle n \rangle /\langle r \rangle = 1$ for all *s*, as predicted by Theorem 1; Models D (squares, translational feedback only) and E (diamonds, both feedbacks) show enrichment increasing with *s*, with Model E systematically below Model D, consistent with the negative second-order correction in $q_1$ (Eq. (30)). The moment closure agrees with simulations within statistical error across the full range of *s* shown

The second-order correction is strictly negative and its magnitude increases with $q_1$ through $n_0$. This provides the analytical basis for the suppression of selective enrichment by transcriptional feedback observed in the Gaussian moment closure and Moran simulations (Figure 2).

#### Generalisation to Nonlinear Feedback Functions

The preceding analysis assumed a linear dependence of translation rate on growth rate. Experimental evidence, however, shows that the relationship between ribosome abundance and growth rate is saturating rather than strictly linear (Dai et al. 2016). We now show that the main result generalises to an arbitrary functional form.

Let the translation rate per mRNA be given by a general smooth function $g(\mu )$ of the growth rate, so that the translation reaction is $m \rightarrow m + {\text {protein}}$ at rate $g(\mu (n)) \cdot m$. The linear case corresponds to $g(\mu ) = k_p + q_2\mu$; a saturating (Michaelis–Menten) form would be $g(\mu ) = k_p + q_2\mu /(K_\mu + \mu )$.

At zeroth order ($s = 0$), the growth rate is $\mu _0$ and the effective translation rate is $g(\mu _0) \equiv k_p^{{\text {eff}}}$, exactly as before. All zeroth-order moments, including the intrinsic covariance $C_{nm}$, are unchanged.

At first order in *s*, we expand $g(\mu _0 + sn) \approx g(\mu _0) + g'(\mu _0)\,sn$, where $g'(\mu _0) = dg/d\mu |_{\mu _0}$. The perturbation calculation of Section 3.2.4 carries through with $g'(\mu _0)$ replacing $q_2$, yielding

31 $$\begin{aligned} \frac{\langle n \rangle }{\langle r \rangle } \approx 1 + \frac{s\,g'(\mu _0)}{d_m + 2\mu _0}. \end{aligned}$$

This is positive whenever $g'(\mu _0) > 0$, that is, whenever the translation rate is an increasing function of the growth rate at the operating point. The shape of the curve determines the magnitude of selective enrichment but not its existence.

For the saturating case $g(\mu ) = k_p + q_2\mu /(K_\mu + \mu )$, the relevant derivative is

32 $$\begin{aligned} g'(\mu _0) = \frac{q_2 K_\mu }{(K_\mu + \mu _0)^2}, \end{aligned}$$

which is strictly positive for $q_2, K_\mu > 0$. The enrichment is weaker than in the linear case (since $g'(\mu _0) < q_2$) but remains present. This is consistent with the computational finding of Ciechonska et al. (2022) that a saturating translation feedback produces selective enrichment (Ciechonska et al. 2022, Supplementary Material, Figure S10).

Theorem 2 (no enrichment under transcriptional feedback) also generalises to this setting, since the mRNA source $\langle h(\mu (n)) \rangle$ remains gene-independent for any transcription function *h*.

These generalisations extend beyond the perturbative level:

##### Theorem 3

(Exact covariance relation for selective enrichment) Consider the two-stage model with an arbitrary fitness landscape $\mu (n)$, an arbitrary transcription function $h(\mu )$, and an arbitrary translation function $g(\mu )$. At steady state,

33 $$\begin{aligned} \langle \mu \rangle \big (\langle n \rangle - \langle r \rangle \big ) = \textrm{Cov}\!\big (g(\mu (n)),\, m_n\big ) - \textrm{Cov}\!\big (g(\mu (n)),\, m_r\big ). \end{aligned}$$

This requires no perturbative expansion, no moment closure, and no linearity assumptions on $\mu (n)$, $h(\mu )$, or $g(\mu )$. Selective enrichment exists if and only if the translation rate covaries asymmetrically with the two mRNAs. In particular, Theorems 1 and 2 are recovered as corollaries: when *g* is constant (no translational feedback), both covariances vanish identically and $\langle n \rangle = \langle r \rangle$.

##### Proof

At steady state, the protein equations with the dilution–selection cancellation (Theorem 1) give $\langle g(\mu (n))\,m_n \rangle = \langle \mu \rangle \langle n \rangle$ and $\langle g(\mu (n))\,m_r \rangle = \langle \mu \rangle \langle r \rangle$. Since $\langle m_n \rangle = \langle m_r \rangle$ for any transcription function $h(\mu )$ (Theorem 2), subtracting yields Eq. (33). The result reduces to Eq. (26) for linear $\mu (n) = \mu _0 + sn$ and $g(\mu ) = k_p + q_2\mu$. $\square$

Overall, the conditions for selective enrichment can be stated without reference to any particular functional form: selective enrichment occurs if and only if the translation rate is an increasing function of the growth rate, and the magnitude of enrichment is controlled by the local slope $g'(\mu _0)$ of this relationship at the basal growth rate.

#### Connection to the Fluctuation–Response Relationship

The Fano factor of the fitness protein at $s = 0$ is

34 $$\begin{aligned} F = 1 + \frac{k_p^{{\text {eff}}}}{d_m + 2\mu _0} = 1 + \beta , \end{aligned}$$

where $\beta = k_p^{{\text {eff}}}/(d_m + 2\mu _0)$ is the translational burst contribution. Let $\alpha = q_2/k_p^{{\text {eff}}}$ denote the growth-rate sensitivity of translation, normalised by the total effective translation rate. Then Eq. (25) can be rewritten as

35 $$\begin{aligned} \frac{\langle n \rangle }{\langle r \rangle } \approx 1 + s\,\alpha \,\beta = 1 + s\,\alpha \,(F - 1). \end{aligned}$$

This has the structure of a fluctuation–response relation (Sato et al. 2003; Lehner and Kaneko 2011): the response to selection (selective enrichment) is proportional to the fluctuation ($F - 1$) in the unperturbed system, modulated by the fraction $\alpha$ of translation that is growth-coupled. If gene expression is Poissonian ($F = 1$), there is no growth-driven enrichment.

**Fig. 3:**
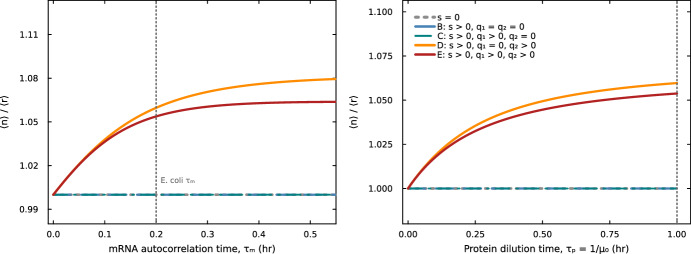
Enrichment ratio $\langle n \rangle /\langle r \rangle$ as a function of two key timescales, at fixed selection strength $s = 0.15$. The left panel shows enrichment vs. mRNA autocorrelation time $\tau _m = 1/(d_m + \mu _0)$ at fixed $\mu _0 = 1$ ${\hbox {hr}}^{-1}$, with $\tau _m$ swept over [0, 0.55] hr. The right panel shows enrichment vs. protein dilution time $\tau _p = 1/\mu _0$ at fixed $d_m = 4$ ${\hbox {hr}}^{-1}$, with $\tau _p$ swept over [0, 1] hr; as $\mu _0$ decreases (slower growth), both $\tau _p$ and $\tau _m$ increase, amplifying enrichment. In both panels, three model variants lacking translational feedback (no selection (grey), growth-coupled selection only (Model B, blue), and selection with transcriptional feedback (Model C, teal)) collapse to $\langle n \rangle /\langle r \rangle = 1$ (overlapping lines at unity; the three curves are indistinguishable in the plot). Only translational feedback (Models D (orange) and E (red)) lifts the ratio above unity, with enrichment increasing monotonically with both $\tau _m$ and $\tau _p$. Model E (with transcriptional feedback, $q_1 > 0$) is visibly below Model D ($q_1 = 0$), consistent with the second-order suppression in Eq. (30). Vertical dotted lines mark typical *E. coli* values ($\tau _m \approx 0.2$ hr, $\tau _p \approx 1$ hr). All curves are computed from the Gaussian moment closure of Appendix D. Parameters: $k_m = 5$ ${\hbox {hr}}^{-1}$, $k_p = 3$ ${\hbox {hr}}^{-1}$, $q_1 = 2$, $q_2 = 3$

This provides a direct analytical connection to the Ohm’s-law-like relationship between selection pressure and enrichment response observed by Ciechonska et al. (2022), where the “conductance” 1/*R* equals $\alpha \beta$. Figure 3 demonstrates this dependence across all model variants: enrichment increases monotonically with both the mRNA autocorrelation time $\tau _m = 1/(d_m + \mu _0)$ (left panel) and the protein dilution time $\tau _p = 1/\mu _0$ (right panel), while models without translational feedback remain at $\langle n \rangle /\langle r \rangle = 1$.

### Concentration-Dependent Fitness

The preceding analysis assumed copy-number fitness: $\mu (n) = \mu _0 + sn$, where the growth rate depends on the total number of fitness protein molecules per cell. In many biological settings, however, the quantity that determines fitness is the protein concentration *n*/*V*, where *V* is the cell volume. In particular, the agent-based simulations of Ciechonska et al. (2022) compute fitness from the concentration of chloramphenicol acetyltransferase, and report concentrations as their output. We therefore extend the framework to concentration-dependent fitness:

36 $$\begin{aligned} \mu (n, V) = \mu _0 + s\,\frac{n}{V}. \end{aligned}$$

In a size-triggered division model, the cell volume grows deterministically within each cell cycle as $V(a) = e^{\mu a}$ (normalising the birth volume to 1), with division occurring at $V = 2$, i.e. at the deterministic interdivision time $T = \ln 2/\mu$. In a steady-state population, whether exponentially growing or maintained at constant size (e.g., in a chemostat or Moran model), the age distribution is not uniform on [0, *T*] but exponential: younger cells are always more abundant because older cells have had more time to be randomly replaced. The steady-state age density is $p(a) = 2\mu \,e^{-\mu a}$ for $a \in [0, T]$, obtained from the balance between ageing and removal with the boundary condition that each division produces two newborn cells. The population average of 1/*V* is therefore

37 $$\begin{aligned} \langle \frac{1}{V} \rangle = \int _0^T 2\mu \,e^{-\mu a}\cdot e^{-\mu a}\,da = 2\mu \int _0^T e^{-2\mu a}\,da = 1 - e^{-2\mu T} = 1 - \tfrac{1}{4} = \frac{3}{4}, \end{aligned}$$

where we used $e^{-\mu T} = 1/2$. Interestingly, $\langle 1/V \rangle$ is independent of the growth rate $\mu$. This is because the exponential growth profile $V = e^{\mu a}$ and the exponential age distribution combine to cancel the $\mu$-dependence exactly.

With concentration-dependent fitness, the selection term in the general moment equation (3) becomes $s\,\textrm{Cov}(n/V, \phi )$ instead of $s\,\textrm{Cov}(n, \phi )$. Strictly speaking, *n* and *V* are not statistically independent: within each cell cycle, the protein number *n* grows together with the volume *V* (newly synthesised proteins accumulate as the cell ages) and both are approximately halved at division, so any age-averaged description that treats them as independent is an approximation. For many eukaryotic genes the bulk transcription rate also scales with cell size (Padovan-Merhar et al. 2015; Sun et al. 2020), which makes the protein concentration *n*/*V* approximately size-independent and further reduces the residual covariance between *n*/*V* and *V*. This size-scaling of transcription does not alter the no-enrichment results: by Theorem 2, a transcription rate that depends on cell volume is a cell-global modulation and cannot by itself produce selective enrichment. A fully resolved description that retains these correlations would require an age- or size-structured agent-based model in the spirit of Thomas (2017); Thomas and Shahrezaei (2021); we adopt the population-level description because it gives closed-form expressions that capture the leading-order effect of cell-cycle averaging. At leading order in *s* (weak selection), the intracellular copy number *n* and cell volume *V* are approximately independent in this averaged sense (*V* is deterministic at fixed growth rate, and stochastic variation in *n* perturbs $\mu$ only weakly). In this regime:

38 $$\begin{aligned} \langle \frac{n}{V} \rangle \approx \langle n \rangle \,\langle \frac{1}{V} \rangle = \frac{3}{4}\langle n \rangle . \end{aligned}$$

Consequently, the concentration fitness $\mu = \mu _0 + s(n/V)$ is equivalent at leading order to copy-number fitness with a rescaled selection strength:

39 $$\begin{aligned} s_{{\text {eff}}} = \frac{3}{4}\,s = 0.75\,s. \end{aligned}$$

Theorems 1 and 2 are preserved under concentration-dependent fitness. For Model B, the cancellation between the dilution term $\langle \mu (n,V)\cdot n \rangle$ and the selection term $s\,\textrm{Cov}(n/V, n)$ holds in the same algebraic form as in the copy-number case: both genes experience the same concentration-dependent growth rate, and the algebraic structure that ensures $\langle n \rangle /\langle r \rangle = 1$ is unchanged. The ratio remains exactly unity for any $s \ge 0$. The same argument extends to Model C with transcriptional feedback only.

For Models D and E with translational feedback ($q_2 > 0$), the first-order enrichment ratio under concentration-dependent fitness is obtained by replacing *s* with $s_{{\text {eff}}}$ in Eq. (25):

40 $$\begin{aligned} {\frac{\langle n \rangle }{\langle r \rangle } \approx 1 + \frac{3\,s\,q_2}{4\,(d_m + 2\mu _0)}.} \end{aligned}$$

The geometric factor 3/4 reflects the cell-cycle averaging of volume variation: the concentration *n*/*V* is largest just after division (when $V \approx 1$) and smallest just before (when $V \approx 2$), and the average over the cycle reduces the effective selection by exactly $25\%$ compared to the copy-number case. All structural conclusions carry through: selective enrichment requires $q_2 > 0$, is independent of $q_1$ at leading order, and scales with the super-Poisson noise $(F-1)$.

When selective enrichment is measured in concentrations rather than copy numbers, the relevant ratio is $\langle n/V \rangle /\langle r/V \rangle$. At weak selection, *n* and *V* are approximately independent, so

41 $$\begin{aligned} \frac{\langle n/V \rangle }{\langle r/V \rangle } \approx \frac{\langle n \rangle \,\langle 1/V \rangle }{\langle r \rangle \,\langle 1/V \rangle } = \frac{\langle n \rangle }{\langle r \rangle }, \end{aligned}$$

and the concentration-measured enrichment ratio equals the copy-number ratio. At stronger selection, corrections from $\textrm{Cov}(n, 1/V)$ appear: cells with more fitness protein grow faster, have larger volumes on average, and thus lower concentrations, producing a negative covariance that slightly reduces the concentration ratio relative to the copy-number ratio. This effect enters at $O(s^2)$ and does not alter the leading-order result.

### Growth Rate Recovery Under Selection

Selective enrichment not only shifts the relative abundance of fitness-gene transcripts but also increases the population mean growth rate. Since $\mu (n) = \mu _0 + sn$, the mean growth rate is $\langle \mu \rangle = \mu _0 + s\langle n \rangle$. Applying Eq. (3) to $\phi = \mu (n) = \mu _0 + sn$ gives

42 $$\begin{aligned} \frac{d\langle \mu \rangle }{dt} = s\frac{d\langle n \rangle }{dt}, \end{aligned}$$

which, after expanding $d\langle n \rangle /dt$ using Eq. (3), contains a strictly positive selection contribution $s^2\,\textrm{Var}(n) > 0$. This is a direct manifestation of Fisher’s fundamental theorem (Fisher 1930): the rate of increase in mean fitness is proportional to the variance in fitness, $\textrm{Var}(\mu ) = s^2\,\textrm{Var}(n)$. For the linear fitness landscape $\mu (n) = \mu _0 + sn$, this means that phenotypic heterogeneity in the fitness gene ($\textrm{Var}(n) > 0$) drives the population mean growth rate upward.

#### Application to Histidine Biosynthesis

In the histidine starvation experiments of Tsuru et al. (2011), the growth rate depends on the intracellular level of the biosynthetic enzyme HisC via a saturating response which can be modelled as

43 $$\begin{aligned} \mu (n) = \mu _0 - s_{{\text {stress}}} + \frac{s_{{\text {stress}}}\,n}{K + n}, \end{aligned}$$

where $\mu _0$ is the unstressed growth rate, $s_{{\text {stress}}}$ is the maximum growth rate penalty imposed by histidine depletion, and *K* is the half-saturation constant. Under full stress with no fitness protein ($n = 0$), the growth rate is $\mu _0 - s_{{\text {stress}}}$; as $n \rightarrow \infty$, the growth rate recovers to $\mu _0$.

In the linear regime ($\langle n \rangle \ll K$), the approximation $n/(K+n) \approx n/K$ maps Eq. (43) onto the standard framework with a reduced basal growth rate $\mu _0 - s_{{\text {stress}}}$ and effective selection strength $s_{{\text {eff}}} = s_{{\text {stress}}}/K$. For Model B (growth-coupled dilution), the steady-state protein mean is $\langle n \rangle = b/(\mu _0 - s_{{\text {stress}}})$, giving the recovery condition

44 $$\begin{aligned} \frac{\langle n \rangle }{K} = \frac{b}{K(\mu _0 - s_{{\text {stress}}})} \gtrsim 1 \end{aligned}$$

for substantial growth rate recovery. To put into words, the population growth rate recovers when the cell’s expression capacity (set by the ratio of the protein production rate *b* to the stressed dilution rate) exceeds the half-saturation constant *K* of the enzymatic pathway. With translational feedback ($q_2 > 0$), selective enrichment further elevates $\langle n \rangle$ above the neutral level, broadening the parameter regime in which recovery occurs.

This result connects phenotypic selection to the emergence of drug resistance: even when phenotypic selection alone cannot fully restore the pre-stress growth rate, by partially recovering growth it extends the window during which cell division occurs under stress, increasing the opportunity for permanent genetic resistance mutations to arise.

### Regulated Expression Bypasses the Need for Translational Feedback

Theorems 1 and 2 assume constitutive gene expression. Many genes, however, are regulated by promoters that stochastically switch between active (ON) and inactive (OFF) states, often called the telegraph model of gene expression. We now show that such regulated expression provides an alternative route to selective enrichment under growth-coupled dilution, without requiring translational feedback ($q_2 > 0$).

Consider a two-state promoter for each gene, with activation rate $k_{\textrm{on}}$ and deactivation rate $k_{\textrm{off}}$. Protein is produced at rate $b_1$ when the promoter is ON and not at all when it is OFF; protein dilution is growth-coupled at rate $\mu (n)$ per molecule, as in Model B. Writing $g_n \in \{0, 1\}$ for the fitness gene promoter state and $g_r$ for the reference, the universal dilution–selection cancellation (Theorem 1) still holds, giving

45 $$\begin{aligned} \frac{d\langle n \rangle }{dt} = b_1\langle g_n \rangle - \langle \mu \rangle \langle n \rangle , \qquad \frac{d\langle r \rangle }{dt} = b_1\langle g_r \rangle - \langle \mu \rangle \langle r \rangle . \end{aligned}$$

At steady state, the enrichment ratio reduces to

46 $$\begin{aligned} \frac{\langle n \rangle }{\langle r \rangle } = \frac{\langle g_n \rangle }{\langle g_r \rangle }. \end{aligned}$$

Enrichment thus arises entirely from a difference in the population-averaged promoter ON fractions. The moment equation for the fitness gene promoter state is

47 $$\begin{aligned} \frac{d\langle g_n \rangle }{dt} = k_{\textrm{on}}(1 - \langle g_n \rangle ) - k_{\textrm{off}}\langle g_n \rangle + s\,\textrm{Cov}(n,\, g_n). \end{aligned}$$

The selection term $s\,\textrm{Cov}(n, g_n) > 0$ because, within a cell, having the fitness promoter ON leads to higher *n* and hence higher growth rate: the promoter state is informative about fitness. For the reference gene, the promoter state $g_r$ is independent of *n* within a cell, so $\textrm{Cov}(n, g_r) = 0$ and $\langle g_r \rangle = k_{\textrm{on}}/(k_{\textrm{on}} + k_{\textrm{off}})$ (the neutral value).

At steady state, $\langle g_n \rangle = (k_{\textrm{on}} + s\,\textrm{Cov}(n, g_n))/(k_{\textrm{on}} + k_{\textrm{off}})$, giving

48 $$\begin{aligned} \frac{\langle n \rangle }{\langle r \rangle } = 1 + \frac{s\,\textrm{Cov}_0(n,\, g_n)}{k_{\textrm{on}}} + O(s^2), \end{aligned}$$

where $\textrm{Cov}_0$ denotes the covariance evaluated at the neutral ($s = 0$) steady state. Computing $\textrm{Cov}_0(n, g_n)$ from the standard telegraph model with dilution rate $\mu _0$ yields

49 $$\begin{aligned} \textrm{Cov}_0(n, g_n) = \frac{b_1\,k_{\textrm{on}}\,k_{\textrm{off}}}{(k_{\textrm{on}} + k_{\textrm{off}})^2\,(\mu _0 + k_{\textrm{on}} + k_{\textrm{off}})}. \end{aligned}$$

Let $F_0 = 1 + b_1\,p_{\textrm{off}}/(\mu _0 + k_s)$ be the Fano factor of the neutral protein distribution, where $p_{\textrm{off}} = k_{\textrm{off}}/(k_{\textrm{on}} + k_{\textrm{off}})$ and $k_s = k_{\textrm{on}} + k_{\textrm{off}}$ is the total switching rate. Substituting into Eq. (48):

50 $$\begin{aligned} \frac{\langle n \rangle }{\langle r \rangle } \approx 1 + s\,(F_0 - 1)\,\tau _g, \end{aligned}$$

where $\tau _g = 1/k_s$ is the promoter autocorrelation time. This result has the same fluctuation–response structure as the translational feedback case (Eq. 35): enrichment is proportional to *s* times the super-Poissonian noise ($F_0 - 1$), modulated by a memory timescale, here the promoter correlation time $\tau _g$ rather than the mRNA lifetime $\tau _m$. In the limit of fast switching ($k_s \rightarrow \infty$), the Fano factor approaches unity, $\tau _g \rightarrow 0$, and Theorem 1 is recovered.

#### Enrichment in the Slow-Switching Regime

**Fig. 4:**
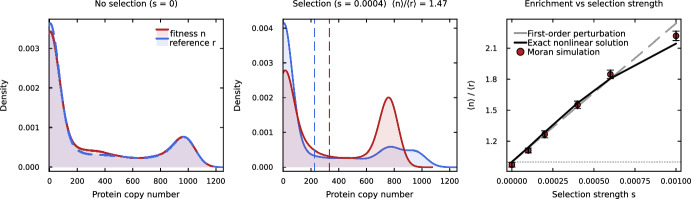
Selective enrichment from regulated expression under growth-coupled dilution, generated entirely by promoter-state memory in the absence of translational feedback. **(a)** Neutral case ($s = 0$): kernel density estimates from Moran-model simulations show that both genes (red: fitness, blue: reference) display identical bimodal distributions, corresponding to OFF (low-*n*) and ON (high-*n*) promoter states under slow promoter switching ($k_{\textrm{on}} = 0.1$ ${\hbox {hr}}^{-1}$, $k_{\textrm{off}} = 0.3$ ${\hbox {hr}}^{-1}$, $b_1 = 1000$ ${\hbox {hr}}^{-1}$, $\mu _0 = 1$ ${\hbox {hr}}^{-1}$). **(b)** Under selection ($s = 4 \times 10^{-4}$): the fitness gene’s upper mode gains weight relative to the lower mode, while the reference gene retains its neutral mode balance. Dashed vertical lines mark the population means $\langle n \rangle$ and $\langle r \rangle$, with the enrichment ratio annotated. **(c)** Enrichment ratio vs. selection strength: first-order perturbation (Eq. 50, grey dashed), exact nonlinear solution (black solid; Appendix F), and Moran simulations ($N = 3000$ cells, $T = 300$ hr per replicate, 15 independent replicates $\pm 1$ SE; red points). The Fano factor of the neutral distribution is $F_0 \approx 537$ and the promoter correlation time is $\tau _g = 1/(k_{\textrm{on}}+k_{\textrm{off}}) = 2.5$ hr

When the promoter switches slowly relative to protein turnover ($k_s \ll \mu _0$), the protein distribution is bimodal: one mode near zero (promoter OFF) and one near $b_1/\mu _0$ (promoter ON). In this regime, selection does not shift a single peak rightward, as in the translational feedback case, but instead changes the relative weights of the two modes. Cells with the fitness gene promoter ON have higher *n*, grow faster, and are overrepresented, increasing the weight of the upper mode for the fitness gene. The reference gene’s promoter, being uncorrelated with fitness, retains its neutral mode balance. The enrichment signature is therefore a shift in mode occupancy rather than a unimodal mean shift, consistent with the bimodal dynamics observed in agent-based simulations of regulated expression under selection (Ciechonska et al. 2022). Figure 4 confirms these predictions: at $s = 0$, both genes show identical bimodal distributions (panel a); under selection, the fitness gene’s upper mode gains weight while the reference gene retains its neutral mode balance (panel b); and the enrichment ratio matches both the first-order perturbation (Eq. 50) and an exact nonlinear solution (Appendix F) across selection strengths (panel c).

The promoter-state selection mechanism ($\textrm{Cov}(n, g_n) > 0$) operates equally under death-driven selection (Section 3.6), where it provides a quantitative enhancement to the enrichment that already arises from constitutive expression. The distinction is that for growth-driven selection, the dilution–selection cancellation eliminates the direct channel, making the promoter-state channel the sole route to enrichment; for death-driven selection, both channels are active.

### Growth-Driven Versus Death-Driven Selection

**Fig. 5:**
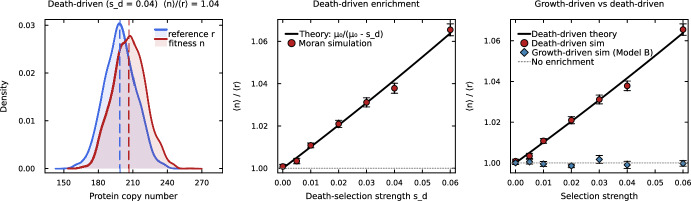
Death-driven versus growth-driven selective enrichment in a protein-only model without translational feedback, illustrating that death-driven selection bypasses the dilution–selection cancellation that suppresses growth-driven enrichment in Model B. **(a)** Protein distributions (kernel density estimates from Moran-model simulations) under death-driven selection ($s_d = 0.04$ ${\hbox {hr}}^{-1}$, linear death rate $\gamma (n) = \gamma _0 - s_d n$): the fitness gene (red) is shifted right relative to the reference (blue), with the population means $\langle n \rangle$ and $\langle r \rangle$ marked by dashed vertical lines and the enrichment ratio annotated. **(b)** Enrichment ratio vs. death-selection strength $s_d$: Moran simulations ($N = 3000$ cells, $T = 200$ hr per replicate, 8 independent replicates $\pm 1$ SE) match the analytical prediction $\mu _0/(\mu _0 - s_d)$ (solid line; see Section 3.6). **(c)** Direct comparison: death-driven selection (red circles) produces enrichment that increases with selection strength, while growth-driven selection with growth-coupled dilution (blue diamonds, Model B) remains at $\langle n \rangle /\langle r \rangle = 1$ due to the exact dilution–selection cancellation (Theorem 1). Parameters: $b = 200$ ${\hbox {hr}}^{-1}$, $\mu _0 = 1$ ${\hbox {hr}}^{-1}$, $\gamma _0 = 0.5$ ${\hbox {hr}}^{-1}$; for the growth-driven curve in panel (c), *s* ranges over the same interval as $s_d$ on the horizontal axis

The preceding analysis establishes that growth-driven enrichment of constitutively expressed genes requires translational feedback ($q_2 > 0$), or alternatively, a source of super-Poissonian gene-specific noise such as promoter switching (Section 3.5). However, experimental studies have reported selective enrichment without invoking any such mechanism. Lasri et al. (2020); Lasri and Sturrock (2021) observed phenotypic selection of MGMT-expressing glioblastoma cells under temozolomide (TMZ) stress, where MGMT confers resistance by repairing DNA damage and thereby reducing cell death. Similarly, the ampicillin model of Ciechonska et al. (2022) (Fig. 5 therein) includes a cell death rate that decreases with intracellular $\beta$-lactamase, rather than a growth rate that increases with the fitness protein. These are examples of death-driven selection, a framework that extends naturally to mammalian cells, where the bacterial coupling between growth rate and translational capacity (Klumpp et al. 2009; Dai et al. 2016) is not established, and to chemotherapy resistance, where drug-sensitive cells are selectively killed while cells that stochastically express higher levels of resistance-conferring proteins persist. Our moment framework reveals why such systems do not require translational feedback.

To see this, we generalise the total fitness to $w(n) = \mu (n) - \gamma (n)$, where $\mu (n)$ is the division rate and $\gamma (n)$ is the death rate. Under the normalised master equation, the general moment equation for the mean fitness protein becomes

51 $$\begin{aligned} \frac{d\langle n \rangle }{dt} = b - \langle \mu (n)\cdot n \rangle + \textrm{Cov}(\mu (n), n) - \textrm{Cov}(\gamma (n), n), \end{aligned}$$

where *b* denotes the total protein production rate. The first selection-related term, $-\langle \mu (n)\cdot n \rangle$, is the dilution penalty: faster-dividing cells dilute their proteins more rapidly. The second, $\textrm{Cov}(\mu (n), n)$, is the growth-driven selection benefit.

The dilution term and the growth selection term sum exactly:

52 $$\begin{aligned} -\langle \mu (n)\cdot n \rangle + \textrm{Cov}(\mu (n), n) = -\langle \mu (n)\cdot n \rangle + \langle \mu (n)\cdot n \rangle - \langle \mu \rangle \langle n \rangle = -\langle \mu \rangle \langle n \rangle . \end{aligned}$$

This is the same cancellation identified in Theorem 1: for purely growth-driven selection ($\gamma = 0$), the selective advantage of faster division is perfectly offset by the faster dilution of proteins. The mean equation closes to $d\langle n \rangle /dt = b - \langle \mu \rangle \langle n \rangle$, and the fitness and reference genes experience identical dynamics, giving $\langle n \rangle /\langle r \rangle = 1$. This exact cancellation is the reason growth-driven enrichment requires translational feedback ($q_2 > 0$): only a growth-rate-dependent translation rate, which multiplies the gene-specific mRNA, introduces an asymmetry between the two genes that survives the dilution–selection cancellation.

For death-driven selection, however, the situation differs. Consider the case where the growth rate is constant ($\mu (n) = \mu _0$) and the death rate decreases with the fitness protein: $\gamma (n) = \gamma _0\,h(n)$ with $h'(n) < 0$. This describes the Lasri TMZ model, where $\gamma (n) \propto {\text {[TMZ]}} / ({\text {[TMZ]}} + s\cdot n)$, and the Ciechonska ampicillin model, where intracellular ampicillin (which induces death) is cleaved by the fitness protein. The moment equation becomes

53 $$\begin{aligned} \frac{d\langle n \rangle }{dt} = b - \mu _0\langle n \rangle - \textrm{Cov}(\gamma (n), n). \end{aligned}$$

The dilution term is now simply $-\mu _0\langle n \rangle$ (constant, gene-independent), and the growth-selection term vanishes because $\mu$ is constant. The only selection contribution is $-\textrm{Cov}(\gamma (n), n)$.

Because the death rate $\gamma (n)$ decreases with *n* (cells with more fitness protein are less likely to die), the covariance $\textrm{Cov}(\gamma (n), n)$ is strictly negative. Therefore $-\textrm{Cov}(\gamma (n), n) > 0$: death-driven selection acts as a positive source term that raises $\langle n \rangle$ above its unselected value. This source term carries no corresponding dilution penalty. Escaping death does not force a cell to dilute its proteins faster; it simply allows the cell and its protein content to persist. The mean equation for the reference protein is $d\langle r \rangle /dt = b - \mu _0\langle r \rangle$ (no covariance term, since $\gamma$ depends on *n*, not on *r*), giving $\langle r \rangle = b/\mu _0$ at steady state. The fitness protein, by contrast, satisfies $\langle n \rangle = b/\mu _0 - \textrm{Cov}(\gamma (n), n)/\mu _0 > \langle r \rangle$, and the enrichment ratio exceeds unity.

This mechanism is purely noise-driven: baseline stochastic gene expression generates a distribution of protein levels, and cells in the low-*n* tail of this distribution die preferentially, shifting the population mean upward. No translational feedback, no explicit mRNA dynamics, and no growth-rate coupling are required. For a linear death rate $\gamma (n) = \gamma _0 - s_d n$ with Poisson protein statistics, the enrichment ratio is $\langle n \rangle /\langle r \rangle = \mu _0/(\mu _0 - s_d)$, analogous to the Mora–Walczak result (Eq. 5) but with the constant dilution rate $\mu _0$ replacing the active degradation rate *d*. This explains why the protein-only models of Lasri et al. (2020); Lasri and Sturrock (2021) and the ampicillin model of Ciechonska et al. (2022) produce selective enrichment without requiring translational feedback, explicit mRNA dynamics, or growth-rate coupling: death-driven selection arises naturally from the overlap between gene expression noise and a survival threshold. Figure 5 confirms these predictions: Moran simulations show that death-driven selection produces enrichment (panel a,b) matching the analytical prediction, while growth-driven selection with growth-coupled dilution (Model B) produces no enrichment at any selection strength (panel c).

When explicit mRNA dynamics and growth-related feedbacks are included in a death-driven model, the enrichment magnitude changes but its existence does not: translational feedback ($q_2 > 0$) enhances selective enrichment by increasing the translational burst size $b = k_p^{{\text {eff}}}\tau _m$ and hence the protein noise, while transcriptional feedback ($q_1$) remains absent at first order in the selection strength, as in the growth-driven case.

Therefore, the requirement for translational feedback ($q_2 > 0$) is specific to growth-driven enrichment of constitutively expressed genes, where the exact dilution–selection cancellation must be broken by a gene-specific mechanism. Death-driven enrichment operates through a distinct pathway (selective removal of low-expressing cells) that bypasses this cancellation entirely. Regulated expression via promoter switching (Section 3.5) enhances death-driven enrichment by providing an additional promoter-state channel ($\textrm{Cov}(n, g_n) > 0$), but is not required for it: the direct survival advantage of high-expressing cells is sufficient. The general moment framework (Eq. 51) unifies all three mechanisms (translational feedback, promoter-state selection, and death-driven selection) and clarifies when each set of minimal ingredients applies.

## Discussion

We have provided an analytical framework for phenotypic selection of fitness-conferring genes, identifying the conditions under which selective enrichment arises across growth-driven and death-driven selection regimes, for both constitutive and regulated gene expression. Computational screening (Ciechonska et al. 2022) identified the minimal ingredients for selective enrichment by testing ten model variants; the analytical framework developed here reveals the mechanistic basis for each requirement, explains alternative routes to enrichment (regulated expression, death-driven selection) within the same framework, and yields quantitative predictions that are difficult to extract from simulation alone.

Table 1 consolidates the model variants analysed above, the simplifying assumptions on which each rests, and the resulting prediction for selective enrichment. The common underlying picture is that growth-coupled dilution exactly cancels growth-driven selection unless a gene-specific source of stochastic memory (intrinsic mRNA fluctuations under translational feedback, or promoter-state fluctuations under regulated expression) persists on a timescale comparable to or longer than the protein dilution time, and unless the selection coupling acts on a rate that multiplies this gene-specific variable. Death-driven selection bypasses the cancellation entirely and therefore needs none of these ingredients.

**Table 1:** Summary of model variants, simplifying assumptions, and analytical predictions for the selective enrichment ratio $\langle n \rangle /\langle r \rangle$ (Figures 2–5)

Model	Production stages	Dilution	Growth/death coupling	Enrichment?	Reference
A (Mora–Walczak)	Protein only	Constant rate *d* (decoupled from growth)	$\mu (n)=sn$	Yes, $d/(d-s)$	Sec. 2.5, Eq. (5)
B	Protein only	Growth-coupled $\mu (n)$	$\mu (n)=\mu _0+sn$	No, $\langle n \rangle /\langle r \rangle =1$ exactly	Sec. 3.1, Thm. 1
C	mRNA + protein	Growth-coupled	Transcriptional feedback only ($q_1>0$, $q_2=0$)	No, $\langle n \rangle /\langle r \rangle =1$ exactly	Sec. 3.2.3, Thm. 2
D	mRNA + protein	Growth-coupled	Translational feedback only ($q_1=0$, $q_2>0$)	Yes, $\approx 1+sq_2/(d_m+2\mu _0)$	Sec. 3.2.4, Eq. (25)
E	mRNA + protein	Growth-coupled	Both feedbacks ($q_1>0$, $q_2>0$)	Yes; slightly suppressed relative to D	Sec. 3.2.4, Eq. (30)
Reg. (telegraph)	Promoter switching $\rightarrow$ protein	Growth-coupled	No translational feedback; ON/OFF promoter	Yes, $\approx 1+s(F_0-1)\tau _g$	Sec. 3.5, Eq. (50)
Death-driven	Protein only (constitutive or telegraph)	Constant $\mu _0$	Death rate $\gamma (n)$ decreases in *n*	Yes, even without feedback or mRNA	Sec. 3.6, Eq. (53)

All predictions are corroborated by Moran simulations

For constitutively expressed genes under growth-driven selection, the no-enrichment theorems (Theorems 1 and 2), the exact covariance relation (Theorem 3), and the enrichment formula (25) together constitute a design rule: selective enrichment requires a growth-rate-dependent feedback that acts on a rate which multiplies a gene-specific stochastic variable, and the resulting response scales with the super-Poisson noise of the unperturbed circuit (Eq. 35). We now discuss the mechanistic basis of these results.

The failure of transcriptional feedback to produce selective enrichment can be understood in terms of timescales and memory. In the two-stage model, each mRNA molecule lives for a characteristic time $\tau _m = 1/(d_m + \mu _0)$ before being degraded or diluted. During its lifetime, each mRNA produces a burst of proteins, and the autocorrelation time of mRNA fluctuations sets a memory window over which a cell retains information about which gene happened to be transcribed more actively. Transcriptional feedback enhances the rate of mRNA production, but this rate is gene-independent: the feedback-enhanced transcription rate $k_m + q_1\mu (n)$ is identical for the fitness gene and the reference gene within the same cell, so that both genes receive the same boost when the growth rate increases. This ensures $\langle m_n \rangle = \langle m_r \rangle$ exactly (Eq. 12), and the downstream protein dynamics inherit this symmetry.

Translational feedback, by contrast, acts at a stage where gene-specific stochastic memory exists. The key timescale is the mRNA autocorrelation time

54 $$\begin{aligned} \tau _m = \frac{1}{d_m + \mu _0}, \end{aligned}$$

which sets the duration over which a protein remains correlated with the mRNA molecule that produced it. In the two-stage model, the intrinsic protein–mRNA covariance is

55 $$\begin{aligned} C_{nm} = \frac{k_p^{{\text {eff}}}\,m_0}{d_m + 2\mu _0} = k_p^{{\text {eff}}}\,m_0\,\frac{\tau _m}{1 + \mu _0\tau _m}, \end{aligned}$$

which is positive and increases with $\tau _m$: longer-lived mRNAs create stronger correlations between a protein and its own mRNA. The fitness protein *n* is correlated with its own mRNA $m_n$ but uncorrelated with the reference mRNA $m_r$ at $s = 0$. The translation rate $(k_p + q_2\mu (n)) \cdot m$ multiplies the gene-specific mRNA copy number *m*, so a cell that has, by chance, more $m_n$ than $m_r$ will translate proportionally more protein *n* when the growth rate is elevated. The resulting excess of protein *n* persists on the much longer protein timescale $\tau _p = 1/\mu _0 \gg \tau _m$, maintaining the selective advantage across multiple mRNA lifetimes and cell division events. This produces a positive feedback loop (more *n* leads to faster growth, which increases translation of $m_n$, which produces more *n*) that does not exist for the reference gene.

The enrichment ratio (Eq. 25) can be rewritten in terms of these timescales as

56 $$\begin{aligned} \frac{\langle n \rangle }{\langle r \rangle } \approx 1 + s\,q_2\,\frac{\tau _m\,\tau _p}{\tau _m + \tau _p}, \end{aligned}$$

since $1/(d_m + 2\mu _0) = 1/(1/\tau _m + 1/\tau _p) = \tau _m\tau _p/(\tau _m + \tau _p)$. The memory effect is thus simply proportional to the harmonic mean of the mRNA and protein timescales, which makes explicit that selective enrichment requires both finite mRNA memory ($\tau _m > 0$, i.e. explicit mRNA dynamics) and translational feedback ($q_2 > 0$) to exploit that memory. In the limit $\tau _m \rightarrow 0$ (instantaneous mRNA, equivalent to a protein-only model), $C_{nm} \rightarrow 0$ and enrichment vanishes, consistent with the failure of Models A and B. In the limit $\tau _p \rightarrow 0$ (infinitely fast dilution), enrichment also vanishes because protein fluctuations are immediately washed out. This dependence on $\tau _m$ is shown in the left panel of Figure 3; the complementary dependence on $\tau _p = 1/\mu _0$ (the protein dilution timescale) is shown in the right panel of Figure 3. Active protein degradation (rate $d_p$) would reduce the effective protein timescale to $\tau _p = 1/(\mu _0 + d_p)$, further suppressing enrichment. Most bacterial proteins are highly stable and are diluted almost entirely by growth rather than active proteolysis, which maximises $\tau _p$ and hence enrichment. Proteins subject to active degradation, such as SsrA-tagged substrates of the ClpXP protease, would be predicted to show weaker selective enrichment, all else being equal. The regulated expression analysis (Section 3.5) reveals a broader principle: any source of gene-specific noise with sufficient memory can drive enrichment, even without translational feedback. In the two-state promoter model, the promoter state $g_n$ is a gene-specific stochastic variable with correlation time $\tau _g = 1/(k_{\textrm{on}} + k_{\textrm{off}})$. Selection enriches for cells where the fitness gene promoter is ON ($\textrm{Cov}(n, g_n) > 0$) but cannot distinguish the reference promoter state ($\textrm{Cov}(n, g_r) = 0$), producing enrichment $\propto s(F_0 - 1)\tau _g$. This unifies the two mechanisms: translational feedback exploits mRNA memory ($\tau _m$), while regulated expression exploits promoter-state memory ($\tau _g$). Both produce enrichment proportional to the super-Poissonian noise $(F - 1)$ times the relevant correlation time. Transcriptional bursting (Golding et al. 2005; Raj et al. 2006) amplifies enrichment in both cases: under translational feedback, bursts inflate the mRNA variance and hence $C_{nm}$; under regulated expression, slow promoter switching directly increases $F_0$ and $\tau _g$. The regulated expression mechanism is closely related to bet-hedging (Balaban et al. 2004; Kussell and Leibler 2005): stochastic promoter switching generates pre-existing phenotypic heterogeneity that selection exploits by differentially favouring one promoter state.

The linear relationship between selection pressure and enrichment response observed by Ciechonska et al. (2022) is a direct consequence of Eq. (25): at weak selection, the ratio is linear in *s* with slope $q_2/(d_m + 2\mu _0)$. The conductance in the Ohm’s law analogy of Ciechonska et al. (2022) is precisely this slope, determined by the noise properties of the gene expression circuit. The connection to the fluctuation–response relationship (Sato et al. 2003; Lehner and Kaneko 2011) is made explicit by Eq. (35): selective enrichment is proportional to the super-Poisson component of protein noise, $F - 1$, where *F* is the Fano factor of the unperturbed ($s = 0$) gene expression distribution. This is an experimentally actionable prediction: one could measure the Fano factor of a gene under non-selective conditions and, given an estimate of the growth-rate coupling $\alpha = q_2/k_p^{{\text {eff}}}$, predict the magnitude of selective enrichment under stress without free parameters. If gene expression were Poissonian ($F = 1$), there would be no growth-driven enrichment regardless of how strong the selection or the translational feedback.

More broadly, the exact relation (26) provides a level of mechanistic insight that simulations and model selection alone cannot. Rewriting it as

57 $$\begin{aligned} \frac{\langle n \rangle }{\langle r \rangle } - 1 = \frac{\overbrace{q_2\,s\,\Delta C}^{{\text {signal}}}}{\underbrace{(\mu _0 + s\langle n \rangle )\langle r \rangle }_{{\text {dilution penalty}}}}, \end{aligned}$$

decomposes selective enrichment into a signal (translational feedback times selection strength times gene-specific covariance asymmetry) divided by a dilution penalty that grows with protein abundance. Growth-coupled dilution is not itself an ingredient for enrichment; rather, it is the biologically realistic feature that creates the exact cancellation (Theorem 1), making enrichment impossible without an additional mechanism that produces a gene-specific covariance asymmetry $\Delta C$ between the fitness and reference mRNAs. The signal-to-penalty decomposition explains what that mechanism must provide: explicit mRNA dynamics are required because $\Delta C$ is built from the intrinsic protein–mRNA covariance $C_{nm}$, which vanishes in the protein-only limit ($\tau _m \rightarrow 0$), and translational feedback ($q_2 > 0$) is required because it is the prefactor of the entire signal.

A positive coupling between growth rate and transcription rate is well established in bacterial physiology, arising from the growth-rate dependence of free RNA polymerase concentration (Klumpp and Hwa 2008; Klumpp et al. 2009). The signal-to-penalty decomposition shows that this feedback not only cannot produce selective enrichment on its own (Theorem 2), but actively hinders it: transcriptional feedback raises the mean protein level ($n_0$ scales with $k_m + q_1\mu _0$), inflating the dilution penalty in the denominator without proportionally strengthening the gene-specific signal in the numerator. The second-order expansion (Eq. 30) makes this precise: the leading $q_1$-dependent correction is $-s^2 q_2 n_0/[\mu _0(d_m + 2\mu _0)]$, which is strictly negative and grows with $q_1$ through $n_0$. Both the exact relation and the Gaussian moment closure confirm that this increased dilution cost dominates any effect $q_1$ may have on the covariance asymmetry $\Delta C$; accordingly, Model E ($q_1 > 0$, $q_2 > 0$) produces weaker enrichment than Model D ($q_1 = 0$, $q_2 > 0$) at all selection strengths, as confirmed by Moran simulations (Figure 2). Importantly, however, the opposition is quantitative, not qualitative: the translational signal is strong enough to overcome the dilution penalty, and selective enrichment persists in the presence of biologically realistic transcriptional feedback. This is consistent with the Bayesian model selection of Ciechonska et al. (2022), which identified Model 7 (translational feedback only) as most likely to produce selective enrichment, with Model 8 (transcriptional and translational feedback) and Model 10 (regulated expression) also producing enrichment. Our analysis shows that the leading-order enrichment ratio is independent of transcriptional feedback strength, and that transcriptional feedback slightly suppresses enrichment at higher orders. The Ciechonska models use a saturating (Michaelis–Menten) fitness function, which can be linearised around the self-consistent operating point to give an effective selection strength $s_{\textrm{eff}}$ that substitutes for *s* in Eq. (25), preserving all structural results (see Appendix D for details).

The extension to concentration-dependent fitness $\mu = \mu _0 + s(n/V)$ (Section 3.3) preserves all structural results but reduces the enrichment magnitude by a parameter-free geometric factor of 3/4. This factor arises from averaging 1/*V* over the cell cycle: exponential volume growth combined with the exponential age distribution of a steady-state population yields $\langle 1/V \rangle = 3/4$, independent of the growth rate. The result is a $25\%$ reduction in enrichment for any gene whose fitness depends on concentration rather than copy number, and connects directly to the agent-based model of Ciechonska et al. (2022), where fitness depends on the concentration of chloramphenicol acetyltransferase. Again, this is a structural prediction: it depends on no model parameters and would be difficult to identify from numerical simulations alone, which would show a quantitative reduction but not directly reveal its origin or universality.

Our analysis uses a continuous dilution approximation rather than explicit cell division with binomial partitioning. In Appendix E, we show that this approximation is exact at the mean level: Theorems 1–3 and all structural conclusions hold under discrete binomial partitioning. Figure 2 confirms these predictions against Moran simulations across the full range of selection strengths, using both the first-order perturbation result and the non-perturbative Gaussian moment closure (Appendix D). Theorems 1 and 2 hold for all *s*, and the exact relation (26) for Model E likewise requires no weak-selection assumption. For stronger selection, higher-order terms in *s* contribute appreciably to the enrichment ratio, but the structural result (selective enrichment of constitutively expressed genes requires $q_2 > 0$) is unchanged.

The growth rate recovery analysis (Section 3.4) connects selective enrichment to a clinically relevant outcome: the population mean growth rate increases at a rate $s^2\textrm{Var}(n)$ (Fisher’s fundamental theorem), and for saturating fitness functions the degree of recovery is governed by the ratio $\langle n \rangle /K$ of protein expression to the enzymatic half-saturation constant. Even partial recovery extends the window for cell division under stress, providing opportunities for permanent genetic resistance mutations to arise. This positions phenotypic selection as a potential early step in the emergence of drug resistance, in both bacterial antibiotic treatment and chemotherapy in mammalian cells.

### Connections to Lineage-Level Fitness Inference

Our analysis adopts a snapshot, population-level perspective in which cells are characterised by their current intracellular state and the population density evolves under Eq. (2). A complementary perspective tracks individual cell lineages. Thomas (2017) formulated an ergodic principle for clonal cell populations, stating that the statistics of cell histories, obtained by tracing a cell in the population back to the ancestor from which it originated, coincide with those of an age-sorted population snapshot. This principle holds for traits that do not affect the division rate, and Thomas and Shahrezaei (2021) analysed the related coupling of gene-expression noise with cell size in agent-based populations. Importantly for the present work, Thomas (2017) also showed that the ergodic principle breaks down precisely when the trait is under selection: for a gene whose expression alters the division rate, the cell-history distribution and the age-sorted population distribution no longer coincide. This breakdown is the lineage-level counterpart of the population-level selective enrichment analysed here, since in both descriptions selection produces a systematic difference between a fitness-conferring gene and a neutrally expressed reference. The breakdown of the ergodic principle under selection is precisely why a genuine population-level description is required: when lineage and snapshot statistics diverge, the population master equation (2) computes the snapshot quantity directly, which is the object relevant to selective enrichment.

This connection is made concrete in Fig. 6 of Thomas (2017), which re-analyses the single-cell genealogical data of Nozoe et al. (2017) for an antibiotic-resistance gene, a streptomycin-resistance gene expressed in E. coli and fused to a fluorescent reporter. In the absence of antibiotic the gene is not under selection, the cell-history and population snapshot distributions agree, and Fisher’s reproductive value is independent of expression level. Under a sub-inhibitory dose of streptomycin, selection acts on the resistance gene, the two distributions diverge, and the reproductive value increases with expression level. The empirical method of Nozoe et al. (2017) quantifies such effects by inferring a fitness landscape and a selection strength directly from population lineage trees, comparing retrospective (ancestral) and chronological lineage statistics. Their selection strength is a lineage-level measure of how strongly reproduction correlates with a phenotypic state, conceptually parallel to the role played by the selection parameter *s* in the growth rate $\mu (n)$ of our framework.

The single-cell time-lapse data of Kiviet et al. (2014) and Nozoe et al. (2017) therefore provide natural datasets against which the predictions of the present framework could be tested. In particular, the gene-specific covariances $\textrm{Cov}(n, m_n)$ and $\textrm{Cov}(n, g_n)$ that drive enrichment in Eqs. (26) and (48) could in principle be extracted from simultaneous time-lapse measurements of mRNA, promoter state, and growth rate in single cells, and compared with the enrichment ratio $\langle n \rangle /\langle r \rangle$ predicted here.

### Model Assumptions, Limitations, and Robustness

The framework developed here is built on a sequence of deliberately minimal models, and it is therefore worth stating the principal assumptions and discussing the robustness of the qualitative conclusions to plausible relaxations of each. The first assumption is spatial homogeneity. Each cell is treated as a well-mixed container of proteins and mRNAs, and the population is treated as a chemostat-like ensemble of identical, non-interacting cells, so that spatial structure such as biofilm geometry, cell-to-cell competition for nutrients, and intracellular localisation of mRNA is ignored. For prokaryotic cells of typical bacterial size and for the freely diffusing proteins considered by Ciechonska et al. (2022), the well-mixed approximation is standard, and spatial fluctuations of mRNA on the timescales relevant to selection are weak. In dense, spatially structured populations such as biofilms or tumour microenvironments, cell-to-cell interactions and spatial gradients of stress can introduce additional selective pressures that our framework does not capture, and our results in such settings should be read as the contribution of intracellular noise to enrichment on top of any spatial heterogeneity.

A second class of assumptions concerns the functional form of the fitness landscape and of the feedbacks from growth rate to gene expression. Theorems 1, 2, and 3 are stated for arbitrary fitness landscapes $\mu (n)$, arbitrary transcription functions $h(\mu )$, and arbitrary translation functions $g(\mu )$, so that none of the structural conclusions rely on linearity. The quantitative slopes such as $q_2/(d_m + 2\mu _0)$ depend on the local derivatives of the feedback functions at the operating point, but the qualitative statement that enrichment requires a gene-specific signal-multiplying feedback is preserved, as discussed in Section 3.2.11.

A third assumption is the use of coarse-grained gene-expression dynamics. The two-stage and three-stage models considered here do not resolve transcriptional bursting, ribosome dynamics, or post-translational modifications, but these additional layers of stochasticity all act on selective enrichment through the Fano factor *F* of the unperturbed protein distribution. Because the fluctuation–response relations (35) and (50) are explicitly proportional to $F-1$, any mechanism that increases super-Poissonian noise, such as transcriptional bursting, slow promoter switching, or coupled transcription and translation, increases enrichment, while any mechanism that suppresses noise relative to Poisson, such as negative autoregulation or fast promoter switching, reduces it. As a particular illustration, a one-stage protein-only model with growth-dependent transcription would have $F = 1$ at $s = 0$ and, consistent with Eq. (35), produce no selective enrichment regardless of the strength of the transcriptional feedback. The Fano-factor decomposition therefore makes our central prediction explicit and robust to the precise functional form of the underlying biochemistry.

A fourth assumption concerns cell size and age structure. As already discussed in Section 3.3, the assumption that *n* and *V* are independent is an age-averaged approximation, and a fully resolved description in the spirit of Thomas (2017); Thomas and Shahrezaei (2021) would retain explicit dependence on cell age or cell volume. For eukaryotic genes in which transcription scales with cell size (Padovan-Merhar et al. 2015; Sun et al. 2020), the protein concentration is approximately size-independent and the leading-order conclusions are unchanged, while for genes that do not scale with size the residual covariance between *n* and *V* enters at $O(s^2)$.

A fifth and final assumption is the use of a population-level description, in which the master equation (2) evolves the population snapshot directly. This master equation is exact at the mean level for both continuous and discrete (binomial) division, as shown in Appendix E, and the population-level means it yields are the quantities of direct relevance to selective enrichment. It does not separately resolve cell-cycle correlations within an individual lineage; for traits whose dynamics are slow relative to a generation the snapshot and lineage descriptions coincide, while for rapidly fluctuating traits or strong mother-to-daughter correlations the two carry genuinely different information, and the appropriate choice is dictated by the question being asked. The main predictions of the framework have been verified against Moran simulations with explicit cell division, as shown in Figures 2–5.

Taken together, these considerations indicate that the central qualitative conclusions of the framework, namely that growth-driven enrichment of constitutive genes requires gene-specific super-Poissonian memory in the form of translational feedback or regulated expression, and that death-driven enrichment does not, are robust to the specific functional form of the gene-expression dynamics. They are statements about which terms survive the dilution–selection cancellation in the population master equation, and they follow from the structure of Eq. (2) rather than from any particular parametric choice.

### Delays between Growth Rate and Fitness Protein Level

Throughout, we have assumed that the cellular growth rate is an instantaneous function of the fitness protein copy number, $\mu = \mu (n)$. In reality, there is a finite delay between an increase in protein abundance and the resulting change in growth rate: ribosome synthesis, metabolic flux adjustment, and biomass accumulation all take time. Kiviet et al. (2014) measured time-resolved cross-correlations between single-cell growth rate and metabolic-enzyme expression in E. coli, and showed that fluctuations propagate between enzyme expression and growth rather than the two adjusting to each other instantaneously. Within our framework, a delay $\tau _d$ between *n* and $\mu$ acts on the selection term $s\,\textrm{Cov}(n, \phi )$ by replacing it with $s\,\textrm{Cov}(n(t-\tau _d), \phi (t))$. For delays short compared to the protein dilution time ($\tau _d \ll \tau _p$), the effect is a small renormalisation of the effective selection strength and does not change any qualitative result. For longer delays, the cross-covariance $\textrm{Cov}(n(t-\tau _d), m_n(t))$ that drives enrichment in Eq. (26) is reduced by a factor approximately $\exp (-\tau _d/\tau _m)$, predicting a quantitative reduction in selective enrichment but no change to the qualitative requirements. The same data of Kiviet et al. (2014) also suggest that growth-rate fluctuations themselves are a source of phenotypic memory that could be exploited by selection; in our framework this would map onto an effective autocorrelation of $\mu$ that augments $\tau _m$ in the fluctuation–response relation (35). A systematic treatment of growth-rate memory is beyond the scope of the present paper but represents a natural direction for future work.

### Broader Applicability and Outlook

The framework developed here applies in principle to any system in which cell fitness varies with the level of a stochastically expressed protein, cells divide and are subject to dilution, and selection acts at the population level. Beyond the bacterial settings of Tsuru et al. (2011); Ciechonska et al. (2022), the death-driven analysis of Section 3.6 is directly relevant to mammalian systems, where the bacterial coupling between growth rate and translational capacity (Klumpp et al. 2009; Dai et al. 2016) is not firmly established but where the selective survival of cells expressing higher levels of resistance-conferring proteins is well documented; examples include MGMT under temozolomide (Lasri et al. 2020; Lasri and Sturrock 2021), P-glycoprotein under cytotoxic chemotherapy, and members of the BCL-2 family under apoptotic stress. The exact covariance relation (33) provides a mechanistic, gene-expression-level counterpart to the lineage-based fitness landscapes measured by the framework of Nozoe et al. (2017), which applies to a broad range of proliferating systems. The framework developed here also speaks to the persistence of bet-hedging phenotypes (Balaban et al. 2004; Kussell and Leibler 2005), where the slow-switching limit of the regulated-expression analysis of Section 3.5 provides a quantitative bridge between bet-hedging and growth-driven phenotypic selection.

Several natural directions for extending the framework follow from the assumptions and approximations discussed above. One is to couple the population master equation to explicit cell-cycle and size-control models in the style of Thomas and Shahrezaei (2021), so as to resolve the cell-cycle corrections that enter our results at $O(s^2)$. Another is to incorporate finite delays in the growth-rate response, in the spirit of Kiviet et al. (2014), building on the analysis of the previous subsection. A third is to extend the framework to multiple interacting fitness genes, where the cross-covariances $\textrm{Cov}(n_i, n_j)$ would drive epistatic enrichment patterns that single-gene experiments cannot detect. Together, these directions offer routes for building on the present framework while preserving its emphasis on closed-form analytical results.

## Data Availability

Data sharing is not applicable to this article as no datasets were generated or analysed during the current study.

## Derivation of the general moment equation

Starting from Eq. (2), multiply both sides by $\phi (\textbf{x})$ and sum over all states:

58 $$\begin{aligned} \frac{d\langle \phi \rangle }{dt}&= \sum _{\textbf{x}} \phi (\textbf{x}) \, \mathscr {L}[P]_{\textbf{x}} + \sum _{\textbf{x}} \phi (\textbf{x})(\mu (\textbf{x}) - \langle \mu \rangle )P_{\textbf{x}} \nonumber \\&= \sum _{{\text {reactions}}} \langle {\text {rate}} \times \Delta \phi \rangle + \langle \mu \cdot \phi \rangle - \langle \mu \rangle \langle \phi \rangle . \end{aligned}$$

For linear selection $\mu (n) = \mu _0 + sn$, the selection term becomes $s(\langle n\phi \rangle - \langle n \rangle \langle \phi \rangle ) = s\,\textrm{Cov}(n,\phi )$.

## Derivation of the Protein–mRNA Covariance

At $s = 0$, the two-stage model for a single gene has effective rates $k_m^{{\text {eff}}} = k_m + q_1\mu _0$ and $k_p^{{\text {eff}}} = k_p + q_2\mu _0$. The steady-state means are $m_0 = k_m^{{\text {eff}}}/(d_m + \mu _0)$ and $n_0 = k_p^{{\text {eff}}} m_0/\mu _0$. The Jacobian of the drift vector $(f_m, f_n) = (k_m^{{\text {eff}}} - (d_m+\mu _0)m,\; k_p^{{\text {eff}}} m - \mu _0 n)$ evaluated at $(m_0, n_0)$ is

59 $$\begin{aligned} A = \begin{pmatrix} -(d_m + \mu _0) & 0 \\ k_p^{{\text {eff}}} & -\mu _0 \end{pmatrix} \equiv \begin{pmatrix} -\gamma & 0 \\ k_p^{{\text {eff}}} & -\mu _0 \end{pmatrix}, \end{aligned}$$

where $\gamma = d_m + \mu _0$. The diffusion matrix (sum of production and turnover contributions) is

60 $$\begin{aligned} D = \begin{pmatrix} 2k_m^{{\text {eff}}} & 0 \\ 0 & 2k_p^{{\text {eff}}} m_0 \end{pmatrix}, \end{aligned}$$

where the diagonal entries are $D_{mm} = k_m^{{\text {eff}}} + (d_m+\mu _0)m_0 = 2k_m^{{\text {eff}}}$ (using $m_0 = k_m^{{\text {eff}}}/\gamma$) and $D_{nn} = k_p^{{\text {eff}}} m_0 + \mu _0 n_0 = 2k_p^{{\text {eff}}} m_0$ (using $n_0 = k_p^{{\text {eff}}} m_0/\mu _0$).

The covariance matrix $\Sigma$ satisfies the Lyapunov equation $A\Sigma + \Sigma A^T + D = 0$. Writing this out component by component:

The (*m*, *m*) entry gives $A_{11}\Sigma _{mm} + \Sigma _{mm}A_{11} + D_{mm} = 0$, i.e.

61 $$\begin{aligned} -2\gamma \,\Sigma _{mm} + 2k_m^{{\text {eff}}} = 0, \qquad \Sigma _{mm} = \frac{k_m^{{\text {eff}}}}{\gamma } = m_0. \end{aligned}$$

The (*m*, *n*) entry gives $A_{11}\Sigma _{mn} + A_{21}\Sigma _{mm} + \Sigma _{mn}A_{22} + D_{mn} = 0$, i.e.

62 $$\begin{aligned} -\gamma \,\Sigma _{mn} + k_p^{{\text {eff}}}\,m_0 - \mu _0\,\Sigma _{mn} = 0, \qquad (\gamma + \mu _0)\Sigma _{mn} = k_p^{{\text {eff}}}\,m_0, \end{aligned}$$

giving

63 $$\begin{aligned} \Sigma _{mn} = \frac{k_p^{{\text {eff}}} m_0}{d_m + 2\mu _0} \equiv C_{nm}. \end{aligned}$$

The (*n*, *n*) entry gives $A_{21}\Sigma _{mn} + A_{22}\Sigma _{nn} + \Sigma _{nn}A_{22} + \Sigma _{mn}A_{21} + D_{nn} = 0$, i.e.

64 $$\begin{aligned} 2k_p^{{\text {eff}}}\,C_{nm} - 2\mu _0\,\Sigma _{nn} + 2k_p^{{\text {eff}}} m_0 = 0, \end{aligned}$$

giving

65 $$\begin{aligned} \Sigma _{nn} = \frac{k_p^{{\text {eff}}}(C_{nm} + m_0)}{\mu _0} = n_0 + \frac{k_p^{{\text {eff}}}\,C_{nm}}{\mu _0} = n_0\!\left( 1 + \frac{k_p^{{\text {eff}}}}{d_m + 2\mu _0}\right) = n_0\,F, \end{aligned}$$

where $F = 1 + k_p^{{\text {eff}}}/(d_m + 2\mu _0)$ is the Fano factor.

## Detailed Perturbative Calculation

We expand all quantities in powers of *s*:

66 $$\begin{aligned} \langle m_n \rangle&= m_0 + s\,m_1 + O(s^2),&\langle m_r \rangle&= m_0 + s\,\tilde{m}_1 + O(s^2), \end{aligned}$$

67 $$\begin{aligned} \langle n \rangle&= n_0 + s\,n_1 + O(s^2),&\langle r \rangle&= n_0 + s\,r_1 + O(s^2). \end{aligned}$$

At $s = 0$, the fitness protein *n* and its own mRNA $m_n$ are correlated, but *n* and the reference mRNA $m_r$ are independent:

68 $$\begin{aligned} \langle nm_n \rangle _0 = C_{nm} + n_0 m_0, \qquad \langle nm_r \rangle _0 = n_0 m_0. \end{aligned}$$

### mRNA Equation at *O*(*s*)

The steady-state mRNA equation is $0 = (k_m^{{\text {eff}}} + q_1 s\langle n \rangle ) - (d_m + \mu _0 + s\langle n \rangle )\langle m_n \rangle$. Substituting the expansions and collecting the *O*(*s*) terms yields:

69 $$\begin{aligned} 0 = q_1 n_0 - \gamma \,m_1 - n_0 m_0, \end{aligned}$$

where $\gamma = d_m + \mu _0$. Here the $q_1 n_0$ comes from the transcriptional feedback ($q_1 s\langle n \rangle$ at zeroth order in $\langle n \rangle$), the $-\gamma \,m_1$ from total mRNA turnover (degradation plus dilution) acting on the first-order mRNA correction, and $-n_0 m_0$ from the growth-enhanced mRNA decay ($-s\langle n \rangle \langle m_n \rangle$ at zeroth order). Solving:

70 $$\begin{aligned} m_1 = \tilde{m}_1 = \frac{n_0(q_1 - m_0)}{\gamma }. \end{aligned}$$

The first-order correction $m_1$ is identical for both genes because the transcription rate depends on the growth rate (via *n*), which is gene-independent. Consequently $q_1$ cannot produce gene-specific effects.

### Fitness Protein Equation at *O*(*s*)

The steady-state protein equation (Eq. 13) at all orders is

71 $$\begin{aligned} 0 = k_p^{{\text {eff}}}\langle m_n \rangle + q_2 s\langle nm_n \rangle - \mu _0\langle n \rangle - s\langle n \rangle ^2. \end{aligned}$$

The *O*(1) balance gives $k_p^{{\text {eff}}} m_0 = \mu _0 n_0$, confirming $n_0 = k_p^{{\text {eff}}} m_0/\mu _0$. Writing this out component by component at *O*(*s*) yields:

72 $$\begin{aligned} 0 = k_p^{{\text {eff}}} m_1 + q_2(C_{nm} + n_0 m_0) - \mu _0 n_1 - n_0^2. \end{aligned}$$

Note that $q_2$ multiplies the zeroth-order cross-moment $\langle nm_n \rangle _0$ directly; there is no $1/\mu _0$ prefactor.

Using $n_0 = k_p^{{\text {eff}}} m_0/\mu _0$, the $n_0 m_0$ terms simplify:

73 $$\begin{aligned} q_2 n_0 m_0 - n_0^2 = n_0(q_2 m_0 - k_p^{{\text {eff}}} m_0/\mu _0) = -n_0 m_0 k_p/\mu _0, \end{aligned}$$

where we used $q_2 - k_p^{{\text {eff}}}/\mu _0 = q_2 - (k_p + q_2\mu _0)/\mu _0 = -k_p/\mu _0$. Thus:

74 $$\begin{aligned} \mu _0 n_1 = k_p^{{\text {eff}}} m_1 + q_2\,C_{nm} - \frac{k_p\,n_0 m_0}{\mu _0}. \end{aligned}$$

### Reference Protein Equation at *O*(*s*)

From Eq. (14), using $\langle nm_r \rangle _0 = n_0 m_0$ (since *n* and $m_r$ are independent at $s=0$):

75 $$\begin{aligned} 0 = k_p^{{\text {eff}}} m_1 + q_2\,n_0 m_0 - \mu _0 r_1 - n_0^2. \end{aligned}$$

The only difference from Eq. (72) is the replacement of $C_{nm} + n_0 m_0$ by $n_0 m_0$: the intrinsic covariance $C_{nm}$ is absent because *n* and $m_r$ are uncorrelated at $s=0$. Using the same simplification:

76 $$\begin{aligned} \mu _0 r_1 = k_p^{{\text {eff}}} m_1 - \frac{k_p\,n_0 m_0}{\mu _0}. \end{aligned}$$

### Enrichment Ratio

Subtracting Eq. (75) from Eq. (72) yields

77 $$\begin{aligned} \mu _0(n_1 - r_1) = q_2\,C_{nm}. \end{aligned}$$

The common terms ($k_p^{{\text {eff}}} m_1$ and $-k_p n_0 m_0/\mu _0$) cancel exactly, leaving only the intrinsic covariance term. Solving for the difference yields:

78 $$\begin{aligned} n_1 - r_1 = \frac{q_2\,C_{nm}}{\mu _0} = \frac{q_2\,k_p^{{\text {eff}}}\,m_0}{\mu _0(d_m + 2\mu _0)}. \end{aligned}$$

Dividing by $$n_0 = k_p^{{\text {eff}}} m_0/\mu _0$$:

79 $$\begin{aligned} \frac{n_1 - r_1}{n_0} = \frac{q_2}{d_m + 2\mu _0}, \end{aligned}$$

yielding the main result Eq. (25). Note that $q_1$ does not appear: it enters both $n_1$ and $r_1$ identically through $m_1$, and cancels in the difference.

## Gaussian Moment Closure

The perturbation expansion of Appendix C is valid when the dimensionless parameter $sn_0/\mu _0$ is small. To extend the analytical predictions to stronger selection, we truncate the moment hierarchy at second order by setting all third cumulants to zero (Gaussian closure). This yields 14 coupled algebraic equations at steady state: 4 for the means ($\langle m_n \rangle$, $\langle n \rangle$, $\langle m_r \rangle$, $\langle r \rangle$) and 10 for the covariances $\sigma _{ij} = \textrm{Cov}(x_i, x_j)$.

The mean equations follow from Eqs. (11)–(14), after applying the $\langle n^2 \rangle$–$\textrm{Var}(n)$ cancellation of Theorem 1. Only two covariances enter the mean equations: $\textrm{Cov}(n, m_n)$ in the equation for $\langle n \rangle$ and $\textrm{Cov}(n, m_r)$ in the equation for $\langle r \rangle$, both multiplied by $q_2 s$. The covariance equations are obtained from the eight intracellular reactions using $d\sigma _{ij}/dt = d\langle x_i x_j \rangle /dt - \mu _i\,d\mu _j/dt - \mu _j\,d\mu _i/dt$, where each reaction contributes $\langle w \cdot \Delta (x_i x_j) \rangle - \mu _i\langle w\cdot \nu \delta _{jp} \rangle - \mu _j\langle w\cdot \nu \delta _{ip} \rangle$. Under the Gaussian closure, the selection term vanishes from the covariance equations. To see why, note that the selection contribution to $d\textrm{Cov}(X,Y)/dt$ is

80 $$\begin{aligned} S_{XY} = s\big [\langle nXY \rangle - \langle n \rangle \langle XY \rangle - \langle Y \rangle \textrm{Cov}(n,X) - \langle X \rangle \textrm{Cov}(n,Y)\big ]. \end{aligned}$$

Expanding $\langle nXY \rangle$ in terms of central moments shows that $S_{XY} = s\,\kappa _3(n, X, Y)$, where $\kappa _3$ is the third cumulant. Setting all third cumulants to zero (the definition of Gaussian closure) therefore eliminates the selection contribution identically, so that selection does not appear explicitly in the covariance equations.

We solve the 14 equations by Newton’s method with parameter continuation from the exact $s = 0$ solution (obtained from the Lyapunov equation of Appendix B), using an adaptive step size in *s* and a numerical Jacobian. The closure preserves three structural properties at every value of *s*: (i) $\langle m_n \rangle = \langle m_r \rangle$ (mRNA equality); (ii) the exact relation (26); and (iii) $\langle n \rangle /\langle r \rangle = 1$ when $q_2 = 0$. Property (iii) follows because the covariance equations decouple from the mean equations when $q_2 = 0$, so the mean equations reduce to those of Model B. At weak selection the closure reproduces the first-order perturbation expansion; at stronger selection it provides a non-perturbative prediction that agrees with Moran simulations (Figure 2).

A notable prediction of the closure is that Model E ($q_1 > 0$, $q_2 > 0$) produces slightly weaker enrichment than Model D ($q_1 = 0$, $q_2 > 0$). This is explained analytically by the second-order correction (Eq. 28): the nonlinear dilution factor $1/(1 + s\,n_0/\mu _0)$ suppresses enrichment when the protein levels are large, and since transcriptional feedback raises the zeroth-order protein level ($n_0 \propto k_m + q_1\mu _0$), it amplifies this dilution penalty without proportionally strengthening the gene-specific signal in the numerator. This suppression is confirmed by the Moran simulations at all selection strengths tested.

## Equivalence of Continuous and Discrete Dilution at the Mean Level

Consider a Moran model in which cell *i* divides at rate $\mu (n_i) = \mu _0 + sn_i$, molecules are partitioned binomially (each molecule independently assigned to one daughter with probability 1/2), and a randomly chosen cell is killed to maintain constant population size. In this discrete-dilution framework, the contribution of division to the population mean of any intracellular quantity $\phi$ decomposes into two terms: (i) the self-dilution of the dividing cell, $-(1/2)\langle \mu (n)\cdot \phi \rangle$, where the factor 1/2 reflects the expected binomial loss; and (ii) the biased replacement, $+(1/2)\langle \mu (n)\cdot \phi \rangle - \langle \mu \rangle \langle \phi \rangle$, where the second daughter replaces a random cell. These combine to give

81 $$\begin{aligned} \frac{d\langle \phi \rangle }{dt}\bigg |_{{\text {div}}} = -\langle \mu \rangle \langle \phi \rangle , \end{aligned}$$

which is identical to the continuous-dilution result. The self-dilution loss and the biased-replacement gain cancel exactly, leaving only dilution at the population-mean growth rate, $-\langle \mu \rangle \langle \phi \rangle$, plus the selection contribution that is already captured by the $\langle n^2 \rangle$–$\textrm{Var}(n)$ cancellation.

Consequently, Theorems 1–3 hold without modification in the discrete-dilution framework, since they follow entirely from the mean equations. In particular, the structural conclusion that selective enrichment requires $q_2 > 0$ and is independent of $q_1$ at leading order is unchanged. The continuous-dilution approximation does, however, affect the variance and covariance equations: binomial partitioning adds extra noise at each division event and alters the effective decay rate of correlations, modifying the single-cell steady-state covariance $C_{nm}$. Since the first-order enrichment ratio depends on $C_{nm}$ (through $q_2 C_{nm}/\mu _0^2 n_0$), the specific numerical coefficient $1/(d_m + 2\mu _0)$ in Eq. (25) is particular to the continuous-dilution model; the Moran model with binomial partitioning would yield a quantitatively different coefficient, though the structural dependence on $q_2$, $d_m$, and $\mu _0$ is preserved. The Moran simulations used for validation in Figure 2 employ continuous intracellular dilution, and confirm the analytical formulas in that setting.

## Exact Nonlinear Solution for Regulated Expression

We derive the nonlinear (all-orders-in-*s*) enrichment ratio for the two-state promoter model with growth-coupled dilution (Section 3.5). The intracellular state of each cell includes the promoter states $g_n, g_r \in \{0,1\}$ (for the fitness and reference genes) and the protein copy numbers *n*, *r*. Protein is produced at rate $b_1 g_n$ (or $b_1 g_r$) and diluted at rate $\mu (n) = \mu _0 + sn$ per molecule. The promoter activates at rate $k_{\textrm{on}}$ and deactivates at rate $k_{\textrm{off}}$, with $k_s = k_{\textrm{on}} + k_{\textrm{off}}$.

### Mean Equations

The universal dilution–selection cancellation (Theorem 1) applies to any production process, giving the mean equations

82 $$\begin{aligned} \frac{d\langle n \rangle }{dt} = b_1\langle g_n \rangle - \langle \mu \rangle \langle n \rangle , \qquad \frac{d\langle r \rangle }{dt} = b_1\langle g_r \rangle - \langle \mu \rangle \langle r \rangle , \end{aligned}$$

where $\langle \mu \rangle = \mu _0 + s\langle n \rangle$. At steady state, $\langle n \rangle /\langle r \rangle = \langle g_n \rangle /\langle g_r \rangle$.

The promoter dynamics for the fitness gene give

83 $$\begin{aligned} \frac{d\langle g_n \rangle }{dt} = k_{\textrm{on}}(1 - \langle g_n \rangle ) - k_{\textrm{off}}\langle g_n \rangle + s\,\textrm{Cov}(n, g_n), \end{aligned}$$

while for the reference gene, $\textrm{Cov}(n, g_r) = 0$ (the reference promoter is independent of the fitness protein within each cell), so $\langle g_r \rangle = k_{\textrm{on}}/k_s$ at steady state.

### Cross-Moment Equation

To compute $\textrm{Cov}(n, g_n) = \langle ng_n \rangle - \langle n \rangle \langle g_n \rangle$, we derive the moment equation for $\langle ng_n \rangle$. Setting $\phi = ng_n$ in the general moment equation, the reaction contributions are:

84 $$\begin{aligned} {\text {Production }} (n \rightarrow n+1 {\text { when }} g_n=1): \quad&+b_1\langle g_n^2 \rangle = b_1\langle g_n \rangle , \nonumber \\ {\text {Dilution }} (n \rightarrow n-1): \quad&-\langle \mu (n)\cdot n \cdot g_n \rangle , \nonumber \\ {\text {Activation }} (g_n\!: 0 \rightarrow 1): \quad&+k_{\textrm{on}}(\langle n \rangle - \langle ng_n \rangle ), \nonumber \\ {\text {Deactivation }} (g_n\!: 1 \rightarrow 0): \quad&-k_{\textrm{off}}\langle ng_n \rangle . \end{aligned}$$

The selection contribution is $s\,\textrm{Cov}(n, ng_n) = s(\langle n^2 g_n \rangle - \langle n \rangle \langle ng_n \rangle )$. The dilution term expands as $-\mu _0\langle ng_n \rangle - s\langle n^2 g_n \rangle$. The $\langle n^2 g_n \rangle$ terms from dilution and selection cancel exactly (another instance of the universal cancellation), yielding

85 $$\begin{aligned} \frac{d\langle ng_n \rangle }{dt} = b_1\langle g_n \rangle + k_{\textrm{on}}\langle n \rangle - (\mu _0 + k_s + s\langle n \rangle )\langle ng_n \rangle . \end{aligned}$$

At steady state, writing $\bar{\mu }= \mu _0 + s\langle n \rangle$:

86 $$\begin{aligned} \langle ng_n \rangle = \frac{b_1\langle g_n \rangle + k_{\textrm{on}}\langle n \rangle }{\bar{\mu }+ k_s} = \frac{b_1\langle g_n \rangle (\bar{\mu }+ k_{\textrm{on}})}{\bar{\mu }(\bar{\mu }+ k_s)}, \end{aligned}$$

where the second equality uses $\langle n \rangle = b_1\langle g_n \rangle /\bar{\mu }$.

### Exact Solution

Substituting Eq. (86) into the covariance and the promoter steady-state equation gives a single nonlinear equation in $\langle g_n \rangle$:

87 $$\begin{aligned} k_s\langle g_n \rangle - k_{\textrm{on}} = s\left[ \frac{b_1\langle g_n \rangle (\bar{\mu }+ k_{\textrm{on}})}{\bar{\mu }(\bar{\mu }+ k_s)} - \frac{b_1\langle g_n \rangle ^2}{\bar{\mu }}\right] , \end{aligned}$$

with $\bar{\mu }= (\mu _0 + \sqrt{\mu _0^2 + 4sb_1\langle g_n \rangle })/2$. This equation is solved numerically for $\langle g_n \rangle$ at each value of *s*, and the enrichment ratio is

88 $$\begin{aligned} \frac{\langle n \rangle }{\langle r \rangle } = \frac{\langle g_n \rangle }{\langle g_r \rangle } = \frac{k_s\langle g_n \rangle }{k_{\textrm{on}}}. \end{aligned}$$

This solution is exact at the level of second moments: no moment closure approximation is needed because the universal cancellation eliminates all terms involving $\langle n^2 g_n \rangle$ or higher. The only approximation is the neglect of correlations between cell volume and intracellular state, which is the same continuous-dilution framework used throughout the paper.

Expanding Eq. (87) to first order in *s* recovers the perturbative result (Eq. 50): $\langle n \rangle /\langle r \rangle \approx 1 + s(F_0 - 1)\tau _g$, where $F_0 = 1 + b_1 p_{\textrm{off}}/(\mu _0 + k_s)$ is the neutral Fano factor and $\tau _g = 1/k_s$. At larger *s*, the exact nonlinear solution captures the saturation of enrichment as the promoter ON fraction $\langle g_n \rangle$ approaches unity, where the perturbative expansion breaks down (Figure 4c).
